# Effects of Host Phylogeny and Habitats on Gut Microbiomes of Oriental River Prawn (*Macrobrachium nipponense*)

**DOI:** 10.1371/journal.pone.0132860

**Published:** 2015-07-13

**Authors:** Tzong-Der Tzeng, Yueh-Yang Pao, Po-Cheng Chen, Francis Cheng-Hsuan Weng, Wen Dar Jean, Daryi Wang

**Affiliations:** 1 Department of Leisure and Tourism Management, Shu-Te University, Kaohsiung, 824, Taiwan; 2 Biodiversity Research Center, Academia Sinica, Taipei, 115, Taiwan; 3 Institute of Fisheries Science, College of Life Science, National Taiwan University, Taipei, 106, Taiwan; 4 Biodiversity Program, Taiwan International Graduate Program, Academia Sinica, Taipei, 115, Taiwan; 5 Department of Life Science, National Taiwan Normal University, Taipei, 116, Taiwan; Shanghai Ocean University, CHINA

## Abstract

The gut microbial community is one of the richest and most complex ecosystems on earth, and the intestinal microbes play an important role in host development and health. Next generation sequencing approaches, which rapidly produce millions of short reads that enable the investigation on a culture independent basis, are now popular for exploring microbial community. Currently, the gut microbiome in fresh water shrimp is unexplored. To explore gut microbiomes of the oriental river prawn (*Macrobrachium nipponense*) and investigate the effects of host genetics and habitats on the microbial composition, 454 pyrosequencing based on the 16S rRNA gene were performed. We collected six groups of samples, including *M*. *nipponense* shrimp from two populations, rivers and lakes, and one sister species (*M*. *asperulum*) as an out group. We found that Proteobacteria is the major phylum in oriental river prawn, followed by Firmicutes and Actinobacteria. Compositional analysis showed microbial divergence between the two shrimp species is higher than that between the two populations of one shrimp species collected from river and lake. Hierarchical clustering also showed that host genetics had a greater impact on the divergence of gut microbiome than host habitats. This finding was also congruent with the functional prediction from the metagenomic data implying that the two shrimp species still shared the same type of biological functions, reflecting a similar metabolic profile in their gut environments. In conclusion, this study provides the first investigation of the gut microbiome of fresh water shrimp, and supports the hypothesis of host species-specific signatures of bacterial community composition.

## Introduction

There has been a long interaction history between gut microbes and their hosts. The intestinal microbes contribute important functions to their hosts, such as fermenting unused energy substrates, training the immune system, preventing growth of pathogenic bacteria, regulating the development of the gut, and producing vitamins for the host [1–5]. Some evidences have shown that these microorganisms also contribute to disease phenotypes, like obesity, vaginosis, and inflammatory bowel disease [6–8]. Moreover, recent studies showed that a certain type of cancer and coronary heart disease are associated with gut microbes [9, 10], and even some animal behavior could be attributed to these microbes [11]. Over the last decades, the increasing demand for aquaculture products has prompted the investigation of bacterial composition in intestine tract and host interaction. Studies have shown that bacteria in the intestines of aquatic animals contribute to the development of host’s immune system and digestive system [12–14]. Some studies of aquaculture animals, including the black tiger shrimp, further confirmed the development of probiotic applications to enhance disease resistance and growth [15]. Through the investigation of model organisms, it is now known that diet, and host genetic divergence might be responsible for the taxonomic composition of the gut microbiome [16–19]. However, the investigation of shrimp gut microbiomes is relatively rare. A study investigated the bacterial composition in the intestine of black tiger shrimp (sea shrimp) under different growth stages, and identified a dominant bacterial group (Proteobacteria) in four developmental stages [20]. Similar microbiome structures were also observed in wild and domesticated adult black tiger shrimp [21].

The *Macrobrachium nipponense* is a non-obligatory amphidromous prawn [22], which is broadly distributed over the East Asian regions (China, Japan, Korea, Vietnam, Myanmar, and Taiwan) [23] and has been introduced in Singapore, Philippines, Uzbekistan, southern Iraq, and Iran [23, 24]. The species has potential for aquaculture because it can be reproduced easily and is highly tolerant of various environments [25]. In fact, *M*. *nipponense* is considered as one of the most important freshwater prawns for aquaculture in China, Korea, and Japan [26]. In addition to the economic value of the oriental river prawn, we found that a group of oriental river prawns dwell in the rivers to complete their life cycle, whereas some populations are found in inland freshwater lakes [25]. In central Taiwan, two different lineages of oriental river prawn were found (Chishan and Shihmen lineages) sharing the same habitats [22]. The divergence in populations and habitats provides a chance to study the host genetics and ecological effect on gut microbiomes. A further investigation on the divergence of gut microbiota can also provide useful information on the management of shrimp aquaculture, as many intestinal microbes are associated with shrimp diseases [27, 28].

Aquatic organisms are in direct and continual contact with the aquatic environment. The complex and dynamic microbiota may have significant effects on their health and development [4], and are involved with host physiology, ecology and evolution [29, 30]. Previous studies on aquatic organisms have shown that host genetic divergence may strongly shape the taxonomic composition of the gut microbiome [4, 5, 16, 17]. However, distinct environments and diets may also cause significant impact on gut microbiota, and obscure their true influence of host species [31]. The freshwater prawns (genus *Macrobrachium*) are omnivorous that primarily consume filament-algae, organic debris, and small aquatic insect or animal carcasses. Cellulose-degrading bacteria play important roles for food degradation in the gut of prawns, especially after feeding on a high cellulose diet. The ecological difference has caused physiological effects on the shrimp (*M*. *nipponense*), with the female size at maturity significantly smaller in the river population than in the lake population [25]. Another fresh water shrimp, *M*. *asperulum* is a landlocked species sharing similar feeding habits with the *M*. *nipponense* lake dwelling group [32]. By making pair-wise comparisons on the oriental river prawns, we aim to investigate the effects of host genetics and habitats on the gut microbiomes taking the advantage of the ecological features of fresh water shrimp.

A microbial community can be monitored using traditional culture dependent techniques. However, since the majority of microorganisms cannot be cultivated, metagenomic analyses which extract DNA information from a microbial community are now commonly applied [4, 5, 16]. The advances in DNA-sequencing technology [33] provide the opportunity to survey complex microbial diversity through the direct sequencing of microbial genes. Among these new techniques, the 454 pyrosequencing technique provides relatively long read lengths and lower error rate, and therefore has been commonly used to study the gut microbiomes on the basis of 16S rRNA sequencing [5, 16, 34]. Using the 454 pyrosequencing technique, we provide the first report on gut bacterial populations in fresh water shrimp (*M*. *nipponense*) dwelling in different environments of distinct water areas. We found evidence supporting the idea that, in oriental river prawns, host genetics had a greater impact on the divergence of gut microbiome than host habitats. The metagenome functions predicted from gut pyrosequencing data were also discussed.

## Results and Discussion

The positive relationship between host genetic and gut microbial divergence suggests an influence of host genotype on the evolution of the microbiome [16, 17, 35]. Moreover, recent studies in mammals also indicated that diet may rapidly shape the gut microbiota [36, 37]. However, there is no related study on fresh water shrimp. Oriental river prawns live in various environments such as river, lake, and estuary [25], and divergent food sources may therefore alter their gut microbiome in response to various digestive strategies [16]. To investigate the association between gut microbiomes and host phylogeny and habitats, we explored the gut microbial community of *M*. *nipponense*, a fresh water shrimp that lives in river and lake habitats, and a landlocked sister species *M*. *asperulum*. We collected a total of six groups of shrimp, one group of *M*. *asperulum* living in the Chishan River, and examples of *M*. *nipponense* living in either river (2) or lake (3) environments (Table 1, S1 Fig). Each microbial community was analyzed by a well-established metagenomic pipeline and the difference between communities was illustrated via statistical models.

**Table 1:** Shrimp sampling profiles.

Sampling Region	Species	Lineage^a^	Abbreviation
Chishan River	*Macrobrachium asperulum*	-	CRA
Chishan River	*Macrobrachium nipponense*	Chishan	CRc
Tahan River	*Macrobrachium nipponense*	Chishan	TRc
Mingte Reservoir	*Macrobrachium nipponense*	Chishan	MLc
Mingte Reservoir	*Macrobrachium nipponense*	Shihmen	MLs
Shihmen Reservoir	*Macrobrachium nipponense*	Shihmen	SLs

^**a**^Lineage identification was performed by PCR and sequencing using shrimp mitochondrial cytochrome oxidase subunit I (COI) gene (data not shown).

### Microbial complexity in oriental river prawn gut

**Fig 1:**
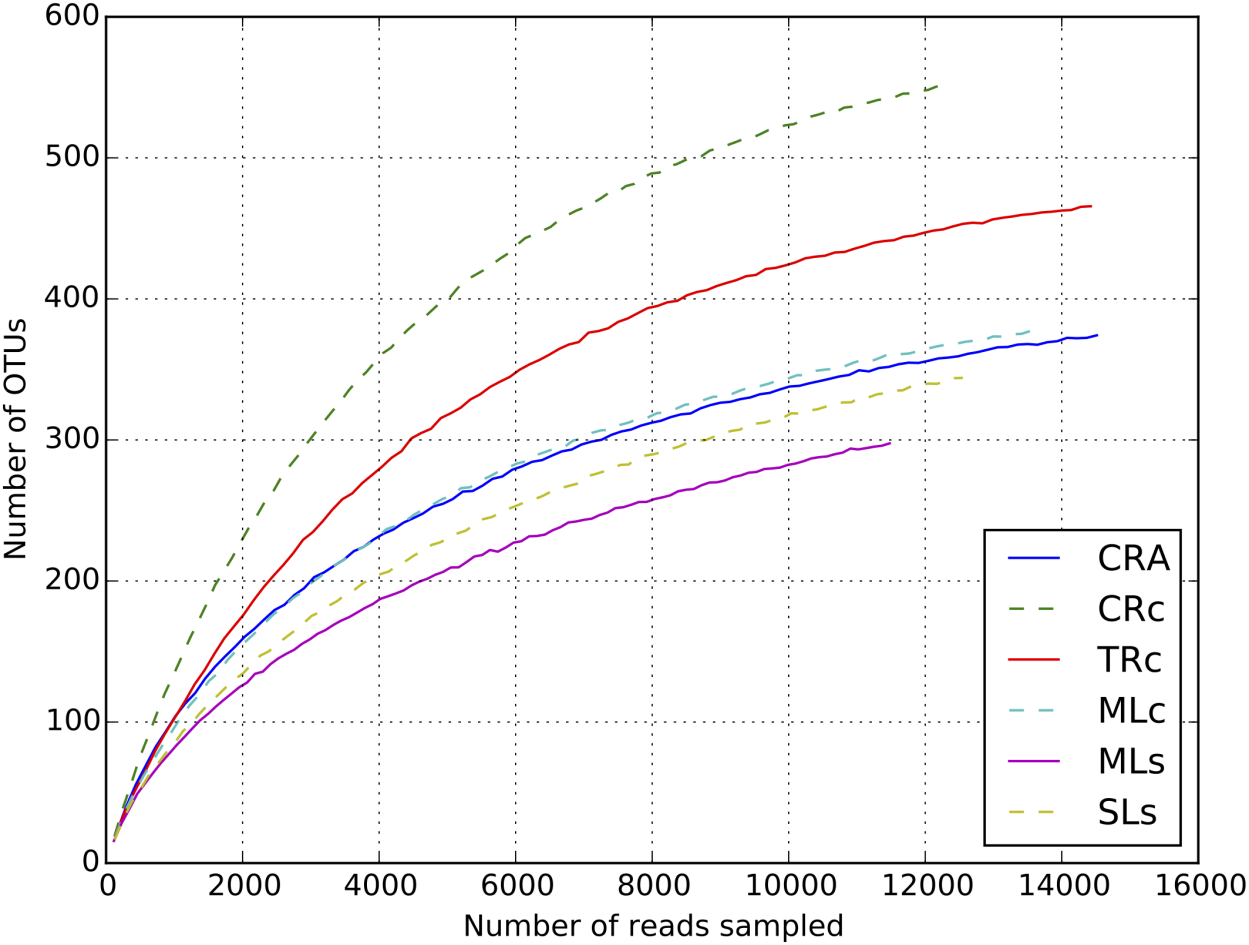
Rarefaction analysis of shrimp gut. Rarefaction curves were calculated from the six libraries at 97% sequence identity of 16S rRNA gene.

To determine bacterial populations in shrimp guts, pyrosequencing of the 16S rRNA gene was employed. After data filtering processes, a total of 68,115 valid reads and 2,987 OTUs were obtained from the six groups of samples (sequences can be downloaded from Bioproject Database with BioProject ID: PRJNA280489). These sequences were assigned to 16 different phyla or groups. Each of the six communities contained reads between 11,479 and 14,514, with OTUs ranging from 422 to 640 (Table 2). The rarefaction curves have approach the saturation plateau (Fig 1), and Good’s coverage estimations revealed that 98.98% to 99.75% of the microbes present were detected in the samples (Fig 1). To estimate the microbial diversity among samples, OTUs of each sample were grouped at an evolutionary distance ≤ 0.03 (97% sequence similarity) for calculation. The results from the Shannon diversity index seemed indicate the samples from rivers (CRc, TRc: range from 4.45–5.07) were slightly higher than the samples from lakes (MLc, MLs, SLs: range from 3.67–3.77), although no significant difference was detected (Wilcoxon rank-sum test). The diversity measured from Chao1 estimator was more evenly distributed, suggesting that the differences in Shannon diversity may have been due to some small populations of microbes (Table 2). It is commonly believed that that the food resources are more complicated in rivers than that in lakes [38], and the diversity data might therefore reflect the complexity of the food source.

**Table 2:** Diversity of shrimp bacterial community analyzed from 16S rRNA pyrosequencing reads.

Sample ID	CRA	CRc	TRc	MLc	MLs	SLs
**No. of sequences**	14514	12285	14426	13681	11479	12550
**No. of OTUs** ^a^	441	640	535	483	422	466
**Coverage**	99.75%	99.63%	99.74%	99.35%	98.98%	99.15%
**Chao1**	451.16	653.10	548.26	545.16	524.82	549.40
**95% Chao 1**	441.09–1646.90	640.10–2343.87	535.14–1775.34	484.13–3890.02	424.29–5045.95	467.63–4740.13
**Shannon**	4.21	5.07	4.45	4.11	3.67	3.77

^a^ Calculations were based on OTUs formed at an evolutionary distance of <0.03 (or 97% similarity)

### Microbial composition in oriental river prawn gut

**Fig 2:**
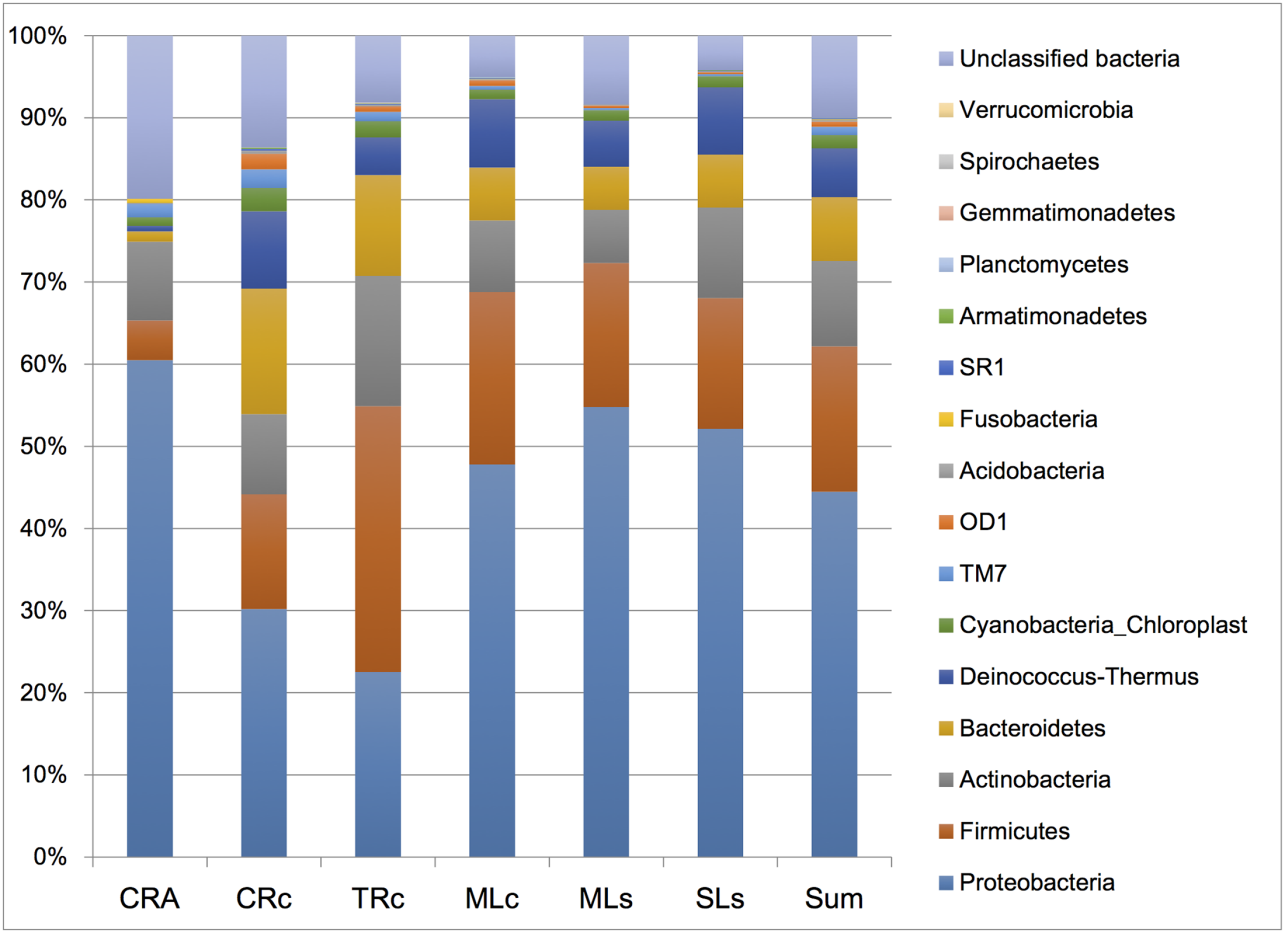
Gut bacterial composition of six libraries in phylum level. Sequences that could not be identified were assigned as “Unclassified bacteria”.

Mothur, an open source software program for analysis and comparison of microbial communities, was used to classify all sequences from phylum to genus, using the default settings [39]. Sixteen different phyla or groups can be identified from these samples. The 16S rRNA profiles are shown at the phylum level in Fig 2. Proteobacteria, which accounted for 23–60% of total populations, is the major dominant phylum in the six groups of oriental river prawn, followed by Firmicutes and Actinobacteria. The microbial composition in *M*. *nipponense* guts are different from those found in black tiger shrimp (in seawater), which are more heavily dominated by Proteobacteria (more than 80%), followed by Firmicutes and Bacteroidetes [20, 21]. As four major groups, Proteobacteria, Actinobacteria, Firmicutes, and Cyanobacteria, are commonly found in some fresh water fish [5, 31, 40], the gut microbiomes of fresh water shrimp seem to have more in common with fresh water fish than with sea shrimp, at least at the phylum level [20, 21].

To further understand the major components of the gut microbial community from different libraries, we focused on phyla that made up more than 1% sequences of the library for comparison (S1 Table). Among the six samples, the MLc and TRc libraries had the largest number of phyla represented (14 phyla), of which Proteobacteria, Firmicutes, Actinobacteria, Bacteroidetes, and Deinococcus-Thermus accounted for 92.7% of the reads in the MLc library and 87.6% of the reads in the TRc library. The MLs, SLs, and CRc libraries contained 11, 12, and 13 phyla, respectively, of which Proteobacteria, Firmicutes, Actinobacteria, Bacteroidetes, and Deinococcus-Thermus accounted for 89.6, 93.7, and 78.6% of the reads from the total sequences. The CRA library from the landlocked species contained the lowest number of phyla (10 phyla). Proteobacteria, Firmicutes, and Actinobacteria were the most dominant phyla and together accounted for 74.9% of the reads. The overall distribution of gut bacterial population in oriental river prawns is different than that in black tiger shrimp, which contained five phylum of which Proteobacteria and Firmicutes accounted for over 90% of the total population [21].

The freshwater prawns (genus *Macrobrachium*) are omnivorous, primarily consuming filament-algae, organic debris, small aquatic insects or animal carcasses [22]. The cellulose-degrading bacteria play important roles for food degradation in the gut of prawns, especially after feeding on a high content of cellulose diet [41]. In the present study, *Actinomyces*, *Anoxybacillus*, *Citrobacter*, *Clostridium*, and *Leuconostoc*, previously reported to degrade cellulose [42] were frequently observed in fresh water prawn (S2 Table). Of these genera, *Citrobacter*, *Anoxybacillus* and *Clostridium* can be observed in all samples, *Actinomyces* was absent in the samples obtained from Chishan River (CRA and CRc), where as *Leuconostoc* only presented in the samples obtained from Mingte Reservoir (MLc, MLs). Since feeding is one of the important factor reflecting the ecological condition in the gut [43], the distribution of cellulose-degrading bacteria may partially reflect the feeding habits.

It is well known that shrimp suffer from many bacterial diseases. For instance, studies have demonstrated that *Acinetobacter*, *Aeromonas*, *Flavobacterium*, *Pseudomonas*, and *Vibrio* can cause bacterial shell disease, early mortality syndrome (EMS), and acute hepatopancreatic necrosis syndrome (AHPNS) [42, 44, 45]. In the present study, the distributions of these genera among the six libraries were surveyed (S2 Table). Sequences assigned to *Acinetobacter* and *Pseudomonas* were relatively low in abundance (0.36–1.76, 0.15–0.47%) in all six libraries. Sequences assigned to *Flavobacterium* were relatively high in five libraries (3.3–8.5%), but low in CRA library (0.5%). *Vibrio*, which is commonly found in marine habitats and animals [46], seemed to be lake group specific, had high abundance, and only appeared in shrimp from lakes (MLc, MLs and SLs libraries, (24.5–32.7%)). On the other hand, *Bacillus*, *Bifidobacterium*, *Enterococcus*, *Lactobacillus*, *Lactococcus*, *Nitrobacter*, *Nitrosomonas*, *Paracoccus*, *Streptococcus* are important probiotics in an aquatic environment [41, 47]. We examined the distribution of these bacteria in the libraries (S2 Table), and found that sequences assigned to *Bacillus* and *Lactobacillus* were frequently observed in all six libraries (0.5–0.96, 1.2–8.9%). Sequences assigned to *Streptococcus* were low in abundance in all six libraries (0.02–0.38%). Sequences assigned to *Paracoccus* were most common in the CRA library (2.2%), and low in the other five libraries (0.02–0.12%). Overall, observed pathogens and probiotics were frequently presented in all six libraries, while some bacteria may reflect the habitat specificity (e.g. *Vibrio*). It’s worth noting that CRA library from the landlocked species seems to enjoy some unique patterns that may reflect its specific adaption in terms of probiotic benefits.

### Comparison of gut microbial composition in shrimp in inter and intra species, and in different living environments

**Fig 3:**
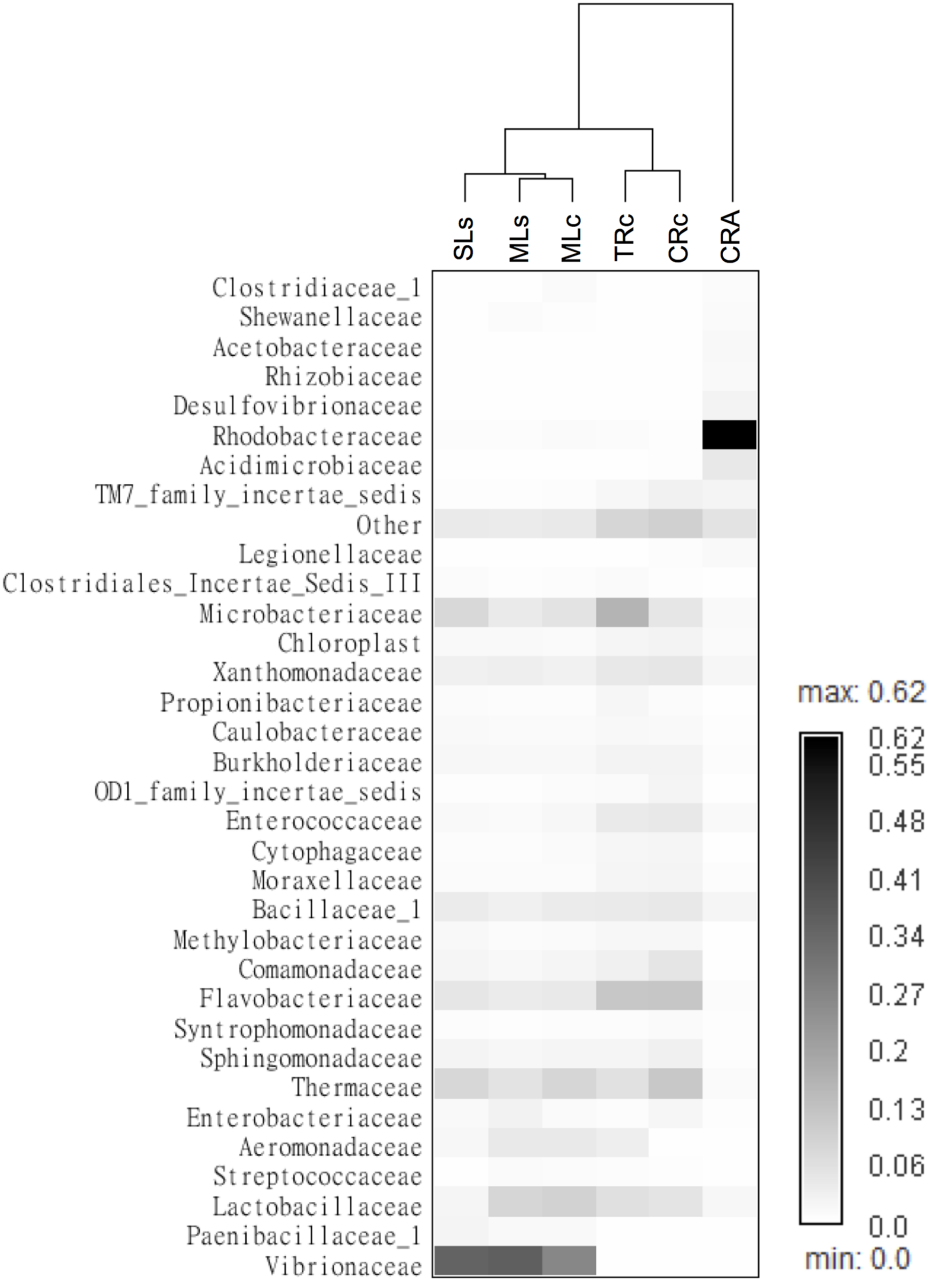
Frequency of OTUs in the shrimp gut microbiomes represented as a heatmap. Shrimp samples were hierarchical clustered based on the correlation matrix of samples calculated by Spearman’s rank. The OTUs were analyzed in family level and only families that made up more than 1% sequences of the library were shown, the rest of the OTUs were grouped as “others”.

Host genetic divergence is one of the major factors determining the gut microbiome [16, 17, 48]. However, studies also revealed the impact of diet and trophic ecology on the microbial structure [16, 31, 37]. To clarify the effects of host genetic and habitats in shrimp gut microbiomes, we performed hierarchical clustering using OTU abundance data in family level (Fig 3). The discussions below were based on the assumptions that two shrimp species consume a similar diet (32), and shrimps from lake or river do exposure on similar microenvironments, respectively. We found the CRA (*M*. *asperulum*) was separated from the other shrimp samples, including Chishan lineage living in rivers and Shihmen lineage living in rivers and lakes. Among the *M*. *nipponense* samples, the samples (MLc, MLs and SLs) obtained from lakes and the samples (CRc and TRc) from rivers formed clusters independently, reflecting the effect of habitats on the divergence of gut microbiomes (Fig 3). Note that the MLc, MLs (Chishan and Shihmen lineages) from Mingte lake and SLs (Shihmen lineages) from Shihmen lake are separated, it is likely that different lakes also contributed more effects on the divergence of gut microbiomes than that from different lineages.

**Fig 4:**
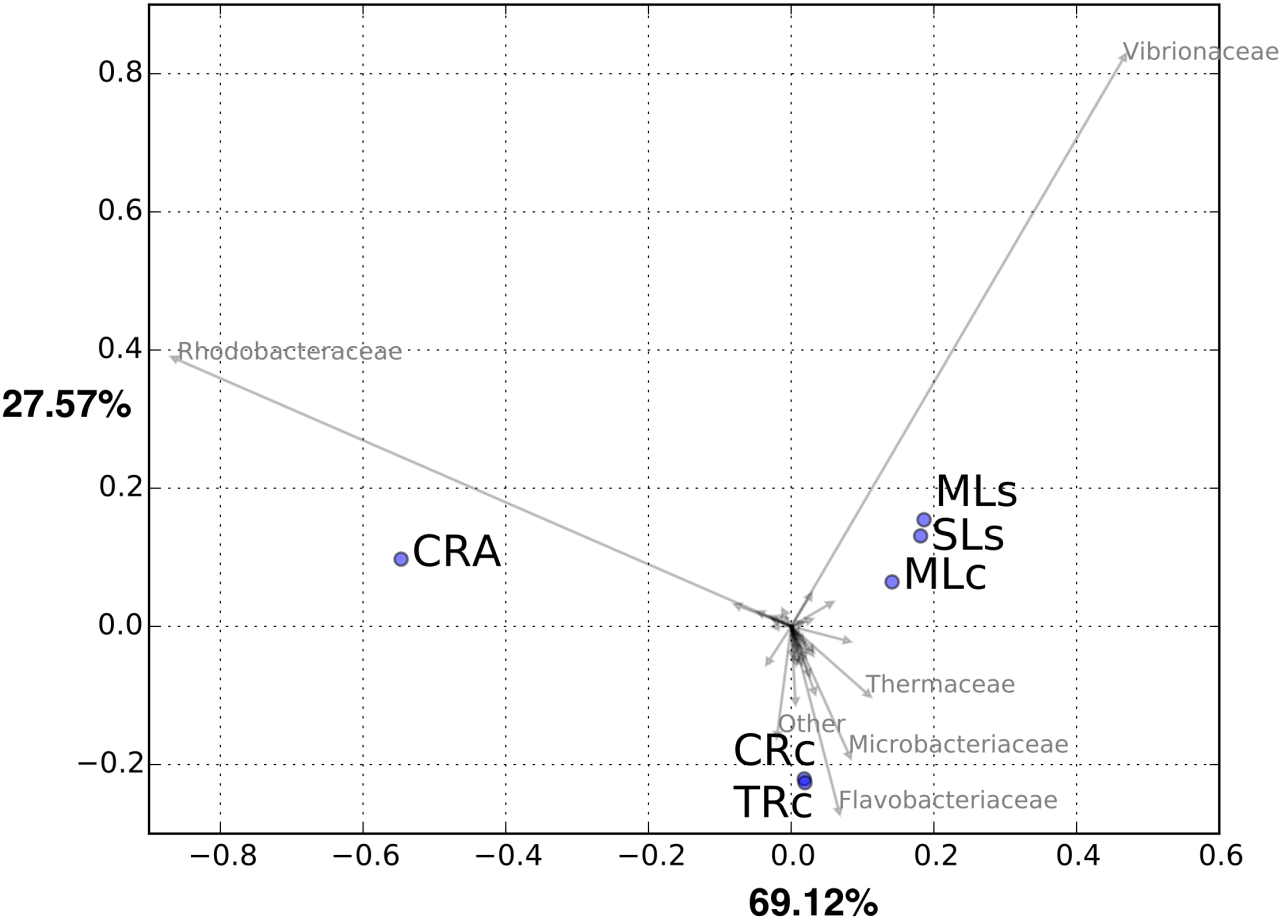
Principal component analysis for six libraries based on OTU abundance in family level. Principal components axes 1 and 2 explained 69.12% and 27.57% of the variance, respectively.

To better visualize the grouping among gut communities of different samples, we plotted the results of a principle coordinates analysis performed in family level (Fig 4). Consistently, the PCA analysis showed that the CRA group separated from the other shrimps, whereas the samples obtained from lakes (MLc, MLs and SLs) and rivers (CRc and TRc) grouped together, respectively. Consistent with Fig 3, PCA plot showed that Rhodobacteraceae and Vibrionaceae appeared to be responsible for the separation of CRA and lake groups, respectively. As *M*. *nipponense* and *M*. *asperulum* were known to share the same feeding habits [22], which may strongly shape the divergence of the gut microbiome [16], our results suggest that host genetic effect seemed to play a more critical role when comparing the gut microbiome of the hosts at the interspecies level. One alternative possibility is that the two shrimp species have different feeding habits and consequently leading to the result. Nevertheless, by comparing the clustering microbial community in two lineages of *M*. *nipponense* (Fig 3), the separation of river and lake samples suggested the host habitats contributed more impact to the divergence than that in hosts of different lineages.

Our data indicated that the host genetic divergence and habitat both play important roles in gut microbial composition. By performing the inter- and intra-species comparison of the shrimps dwelling in river and lake, we found a more critical contribution of host genetic effects on the divergence of shrimp gut microbiomes. One of the debates surrounding the gut metagenome is that the microbiome is highly variable both within a single subject and between different individuals [18, 36, 49–51]. For example, the studies in humans [36, 49] and other animals [18, 50, 51] have demonstrated that there are large variations in microbial composition when comparing individuals from different populations and from different environments. Moreover, the study in chicken cecal microbiota showed that even under carefully controlled conditions large variations in microbial composition can occur, the presence and function of core microbiome remain difficult to analyze [51]. In our experimental design, we pooled five shrimps to reduce the individual bias, moreover, we applied the function analysis to seek for more evidential support for the divergence of gut microbiomes (shown below).

### Shared and unique microbial populations

**Fig 5:**
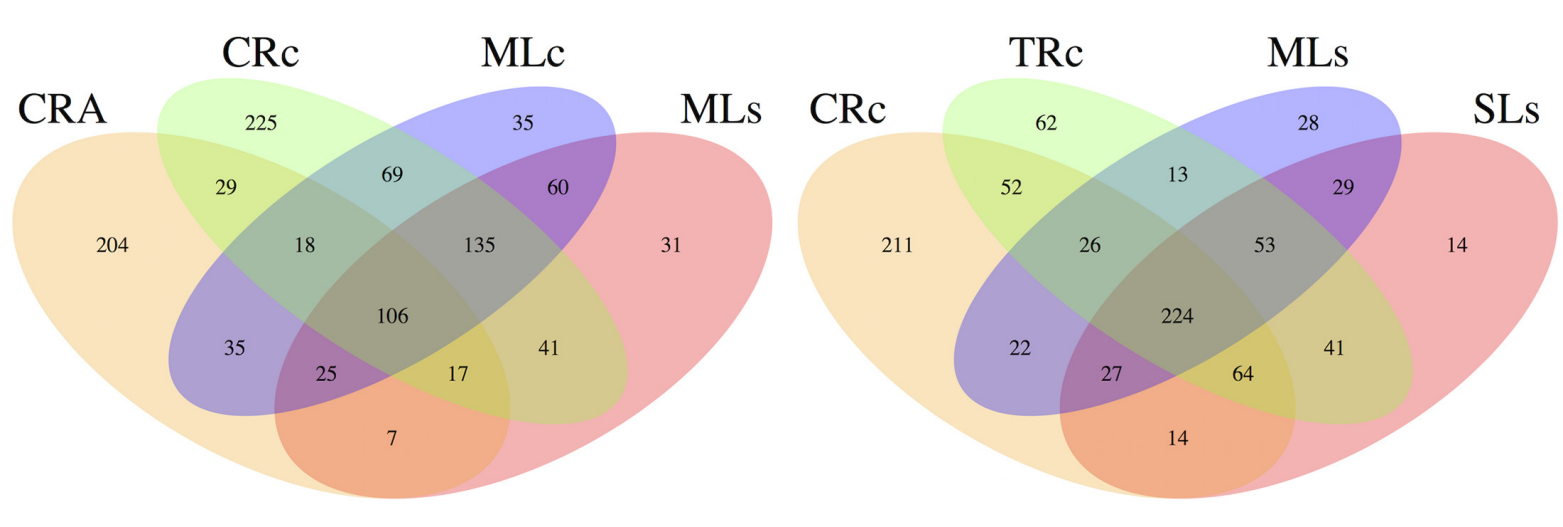
Shared OTUs analysis of sequencing reads obtained from six libraries. A: The Venn diagram represents the share and unique OTUs between shrimps *M*. *nipponense* and *M*. *asperulum*. B: The Venn diagram represents the share and unique OTUs between shrimps dwelling in lakes and/or rivers.

We first revealed the sharing of the gut microbial composition in shrimp of two populations (Chishan and Shihmen) and the sister species. The Venn diagram showed that these two shrimp species (*M*. *nipponense* and *M*. *asperulum*) from the same river shared 170 OTUs (38.5% of CRA, 26.6% of CRc) in the libraries while the two lineages from the same lake shared 326 OTUs (67.5% of MLc, 77.3% of MLs) (Fig 5A). The data showed that the gut microbial community of shrimps of different species shared less OTUs compared to that of shrimp of different lineages. Secondly, by comparing the gut microbial composition of shrimps (*M*. *nipponense*) collected from rivers and lakes, the sharing OTUs were 299 (46.7% of CRc, 70.9% of MLs) and 382 (71.4% of TRc, 82.0% of SLs), while samples from two rivers shared 366 (57.2% of CRc, 68.4% of TRc), and samples from two lakes shared 333 OTUs (78.9% of MLs, 71.5% of SLs), respectively (Fig 5B). Overall, the hosts of different species shared less OTUs compared with the number in all the other pairs, and the results are consistent with the hierarchical clustering (Fig 3) and PCA analysis (Fig 4). It is worth noted that in addition to the bacteria frequently observed in aquatic animal guts (such as *Cryobacterium*, *Microbacterium*), the pathogens (*Acinetobacter*, *Pseudomonas*, *Flavobacterium*), cellulose-degrading bacteria (*Citrobacter*, *Anoxybacillus*, *Clostridium*) and probiotics (*Bacillus*, *Lactobacillus*, *Streptococcus*, *Paracoccus*) were annotated as core microbiome in this study (S2 Table), these bacteria may reflect the functional roles in immune and digestion system in shrimp gut.

**Fig 6:**
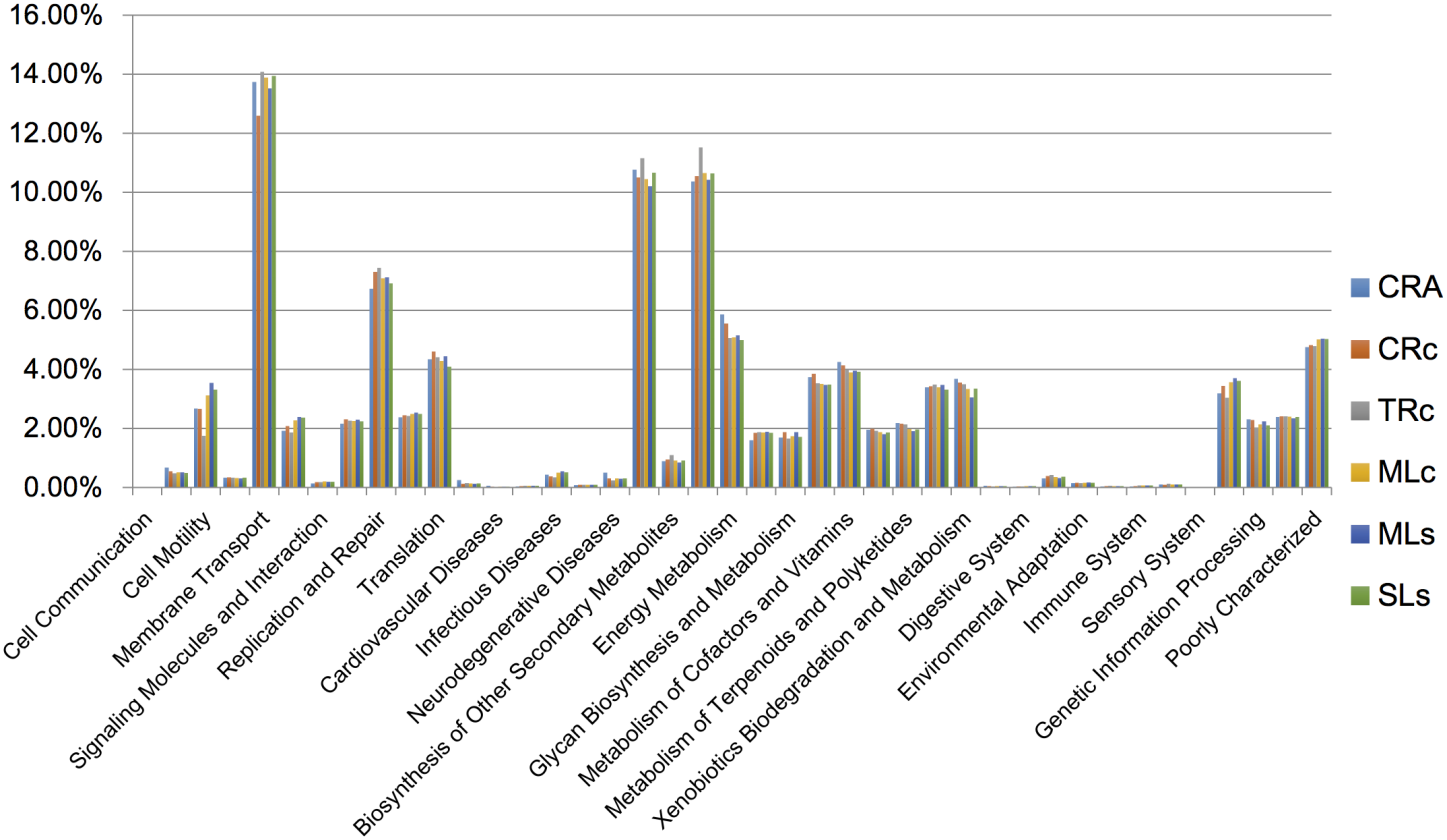
The Predicted relative abundance of KEGG ortholog groups from shrimp gut. The metagenomic function was predicted by PICRUSt base on 16S rRNA gene.

To further investigate the functional divergence among the gut microbiomes, we used PICRUSt (Phylogenetic Investigation of Communities by Reconstruction of Unobserved States) to predict metagenome function based on 16S rRNA sequences [52]. PICRUSt prediction suggested that environmental information processing (e.g. membrane transport) and metabolism (e.g. carbohydrate metabolism, amino acid metabolism) were the dominant functional categories in the fresh water shrimp gut. However, even though there was low overlapping of OTUs between CRA and CRc (Fig 5A) it seems no functional difference was observed between these two groups (Fig 6). Due to limited sample size for the functional prediction (n = 1), statistical support remain limited to rule out an alternative possibility. Nevertheless, the results from functional prediction might imply that *M*. *asperulum*, *M*. *nipponense*, and shrimps obtained from different habitats share the same type of gut metagenome function, reflecting a similar metabolic profile in their gut environments. Therefore, it is reasonable to infer that the divergence of gut microbiomes between CRA and CRc is primarily due to the host genetic effect associated with their evolutionary history. The habitats contributed relatively less, since gut microbial community in shrimps of different habitats still maintain a similar metagenome function. Our results are consistent with the case found where gut microbiomes harbored by great apes reflect the phylogeny of their hosts [48, 53], where as some recent cases reported the convergence of gut microbial communities harbored by great apes and myrmecophagous mammals [18, 37]. In conclusion, this study provides the first evidence for host-specific patterns in gut microbiome of fresh water shrimp, in agreement with previous study found in vertebrates [5, 35, 36, 51, 54]. Investigations using more individuals on various host derived factors will be useful to clarify the debate whether each organism harbors a unique microbiome [37, 55].

## Materials and Methods

### Sample collection and DNA extraction

The study aimed to investigate the divergence of shrimp gut microbiome and its association with living environments of distinct water areas. We collected samples of the same growth stage from two types of habitats: rivers and lakes (reservoirs), inclusive of Tahan River, Chishan River, Shihmen Reservoir (lake) and Mingte Reservoir (lake) (S1 Fig). For each group, five male shrimps of similar weights were collected for dissection. Shrimp weight ranged from 1.7–4.4 g (S3 Table), no significant difference among groups was found using ANOVA test. All shrimps were collected between November and December 2012 and stored at -20°C until workup. To avoid gender bias, only male shrimps were used. Samples were divided into six groups according to habitats, species, and lineages (Table 1). Each microbial community was analyzed by the well-established metagenomic pipeline described below and the comparison between communities was illustrated via statistical models.

Shrimps were aseptically washed with 70% EtOH and instruments were flame sterilized prior to dissection. After dissection, the contents of entire intestine from five shrimps were pooled for metagenomic sequencing. Samples were kept in 1.75mL eppendorf tubes with 0.1mL distilled water and homogenized by 1.5mL disposable pestles (SSI-plastics, USA). The bacterial DNA form homogenized intestines were extracted by QIAamp DNA Stool Mini Kit (*Qiagen*, *GmbH*, *Hilden*, *Germany*) and quantified by *Qubit 2*.*0* Fluorometer (Invitrogen, Life technologies, Carlsbad, CA., USA). Subsequent analysis was conducted with DNA mixtures containing equivalent amounts of DNA from the pooled samples. All procedures were performed in laminar flow cabinet.

### 16S rRNA amplicon preparation and 454 pyrosequencing

The first two variable regions (V1 and V2) of the small subunit rRNA gene were amplified using universal eubacterial primers. The forward primer 27F (5’-AGAGTTTGATCMTGGCTCAG-3’) and the reverse primer 355R (5’-GCTGCCTCCCGAGGAGT-3’) [56, 57] were complemented with 454 adapters and sample specific ten-nucleotide barcodes (S4 Table) to allow multiple samples to be analyzed in parallel on a single 454 picotiter plate. The pooled DNA was amplified with PCR (Taq DNA Polymerase 2x Master Mix Red, Biomol, GmbH, Germany) under the following running conditions: initial denaturation at 95°C for 5 min, 35 cycles of 1 min at 95°C, 45s at 55°C, 1 min at 72°C, and a final elongation step for 7 min at 72°C. PCR products were confirmed using 1.5% agarose gel electrophoresis and were subsequently isolated from the gel and purified using Gel/PCR DNA Fragments Extraction Kit (Geneaid, Geneaid Biotech Ltd., Taiwan). The mixed pool of PCR products was sequenced at Genomics BioSci & Tech Co. in Taiwan, using the Roche/454 GS Junior platform (Branford, CT, USA). Considering the relatively low reads number of 454 GS Junior platform (about 70,000 amplicons per run), six samples were split into two runs for sequencing.

### Pyrosequencing analysis

Raw amplicon sequences from 454 pyrosequencing were first demultiplexed and filtered by software Mothur [39]. The criteria for filtering were read length (minimum of 150 and maximum 450 bp), sequence quality score (minimum of 30), number of errors in the barcode (maximum of 1) and number of errors in the primer (maximum of 2). Barcode and primer sequences were removed from 5’ and 3’end, chimeras were checked and removed using the uchime_ref command in USEARCH [58]. After filtering and trimming processes, reads with an average length of 288bp among all samples were used for downstream metagenomic analyses.

An UPARSE pipeline was used to cluster preprocessed reads into operational taxonomic units at 0.97 similarity, the OTUs with only one read were removed from analysis. The bacterial 16S rRNA reference alignment sequences was exported from RDP, and OTUs were assigned into taxonomy hierarchy by Mothur (Classify.seqs) based on the reference sequences from RDP (version 9) [59]. To evaluate the fraction of species sequenced in each sample, rarefaction curves were generated by using fasta_rarify command in USEARCH. The microbial diversity was analyzed using Mothur based command. The coverage index was calculated by 1-(n/N), where n is the number of phylotypes and N is the total numbers of reads.

A graphical environment for matrix visualization and cluster analyzer (Gap) [60] was used to generate hierarchical clustering and to present the abundance of grouped OTUs with a heat map. Spearman’s rank was used to generate correlation matrix among both samples and grouped OTUs. Average-linkage was then used to calculate hierarchical clustering among samples based on the correlation matrix. In order to reduce noise within the data, only OTUs that made up more than 1% sequences of the library was shown in figures, the rest of the OTUs were grouped as “others”. Since reads that were assigned to unclassified were relatively high at genus level (~45%), data at family level (unclassified ratio ~29%) was chosen for analysis. OTUs that cannot be assigned in family level were excluded for plot.

To perform a parallel comparison, we conducted principal component analysis (PCA) using OTUs in family level with the software package in MATLAB environment. To reveal the sharing OTUs among samples, the Venn diagram was drawn with VennDiagram package in R environment (http://www.r-project.rog). PICRUSt [http://picrust.github.io/picrust/] [52] was used to predict metagenome function base on 16S rRNA gene. The OTUs and abundance data obtained from above mentioned methods were used to estimate the KEGG orthology groups (KOs).

## Supporting Information

S1 Fig Sampling locations in Taiwan (Abbreviations in Table 1). (TIFF)

S1 Table Relative abundance of the shrimp gut microbes in phylum that made up more than 1% sequence of the library. (DOCX)

S2 Table Relative abundance of OTUs of six libraries obtained from shrimp gut. (PDF)

S3 Table Weight distribution of collected shrimps. (DOCX)

S4 Table Primer list of 16S rRNA gene amplicon preparation. (DOCX)
