# Supplementary figures and images for: Effects of Host Phylogeny and Habitats on Gut Microbiomes of Oriental River Prawn (Macrobrachium nipponense)
Source: PLoS One. 2015 Jul 13;10(7):e0132860. doi: 10.1371/journal.pone.0132860 (PMC4500556; doi:10.1371/journal.pone.0132860)

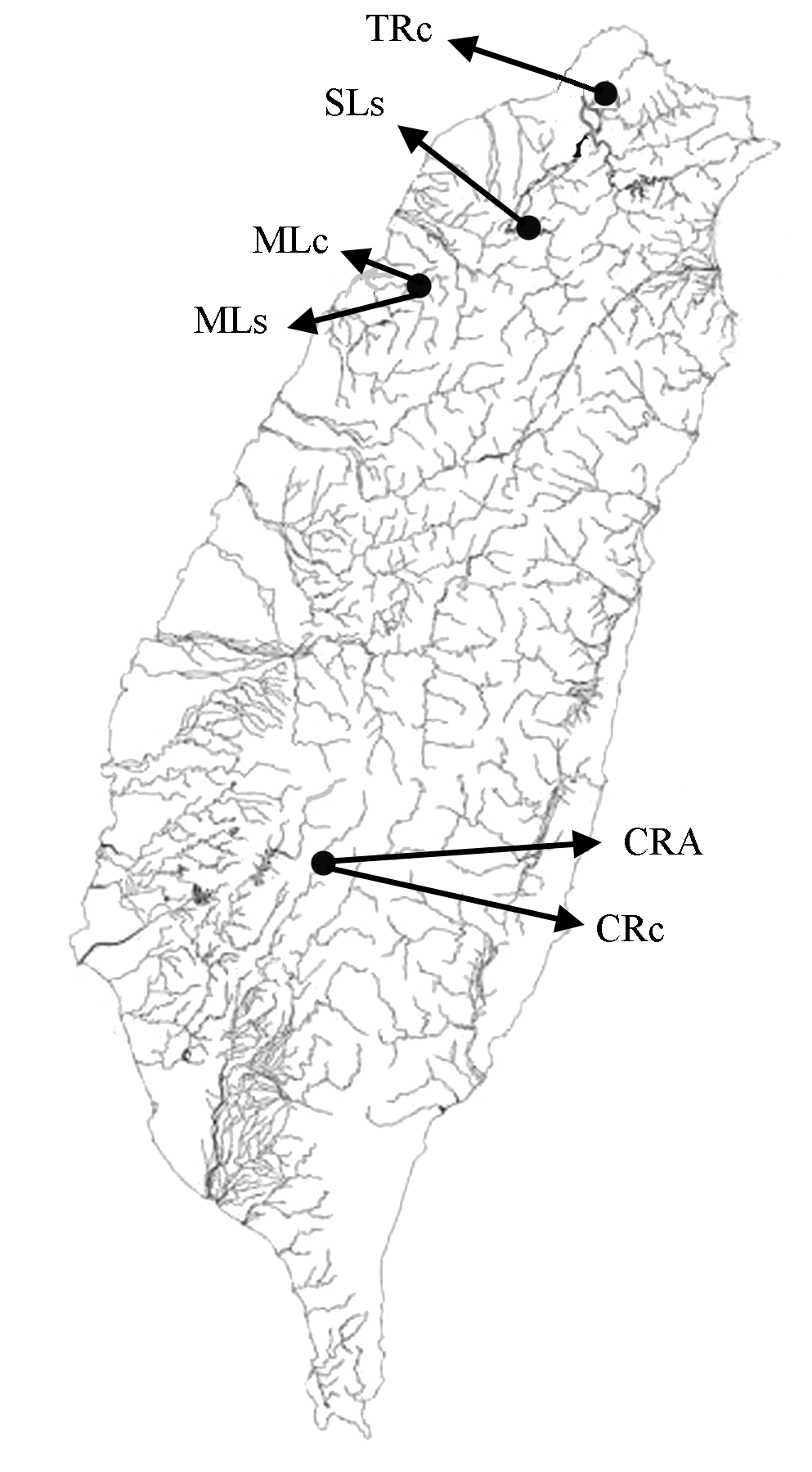

Supplement: S1 Fig — (TIFF) [file pone.0132860.s001.tiff]
