# Supplementary material for: Effects of Host Phylogeny and Habitats on Gut Microbiomes of Oriental River Prawn (Macrobrachium nipponense)
Source: PLoS One. 2015 Jul 13;10(7):e0132860. doi: 10.1371/journal.pone.0132860 (PMC4500556; doi:10.1371/journal.pone.0132860)
Supplement: S1 Table — (DOCX) [file pone.0132860.s002.docx]

**S1 Table. Relative abundance of the shrimp gut microbes in phylum that made up more than 1% sequence of the library.**

|  | CRA | CRc | TRc | MLc | MLs | SLs |
| --- | --- | --- | --- | --- | --- | --- |
| Proteobacteria | 48.4 | 14.0 | 32.4 | 21.0 | 17.6 | 16.0 |
| Firmicutes | 9.6 | 9.8 | 15.9 | 8.7 | 6.5 | 11.0 |
| Actinobacteria | 1.3 | 15.3 | 12.3 | 6.4 | 5.3 | 6.4 |
| Bacteroidetes | - | 9.4 | 4.6 | 8.3 | 6.0 | 8.2 |
| Deinococcus-Thermus | - | 2.9 | 2.0 | 1.1 | 1.3 | 1.4 |
| Cyanobacteria_Chloroplast | - | 2.3 | 1.1 | - | - | - |
| TM7 | - | 1.9 | - | - | - | - |
