# Supplementary material for: Effects of Host Phylogeny and Habitats on Gut Microbiomes of Oriental River Prawn (Macrobrachium nipponense)
Source: PLoS One. 2015 Jul 13;10(7):e0132860. doi: 10.1371/journal.pone.0132860 (PMC4500556; doi:10.1371/journal.pone.0132860)
Supplement: S2 Table — (PDF) [file pone.0132860.s003.pdf]

S2 Table. Relative abundance of OTUs of six libraries obtained from shrimp gut.

OTUID (Genus Name)	CRA	CRc	TRc	MLc	MLs	SLs
OTU_1 (unclassified)	0.00000000	0.00000000	0.17808124	0.02214750	0.01733600	0.04916335
OTU_2 (unclassified)	0.11678379	0.00000000	0.00000000	0.00153498	0.00043558	0.00079681
OTU_3 (unclassified)	0.12608516	0.00016280	0.02384583	0.00438564	0.04852339	0.00764940
OTU_4 (Stenotrophomonas)	0.01226402	0.03882784	0.02987661	0.02689862	0.03066469	0.02996016
OTU_5 (Thermus)	0.00516742	0.08131868	0.03854152	0.06037570	0.03806952	0.05609562
OTU_6 (unclassified)	0.12739424	0.00040700	0.00000000	0.00233901	0.00087116	0.00039841
OTU_7 (unclassified)	0.00000000	0.00333740	0.05046444	0.01739639	0.01210907	0.02358566
OTU_8 (unclassified)	0.00082679	0.03785104	0.01226951	0.00979461	0.00766617	0.00661355
OTU_9 (Vibrio)	0.00000000	0.00000000	0.00006932	0.24581536	0.32746755	0.32000000
OTU_10 (Flavobacterium)	0.00089569	0.06959707	0.04041314	0.02814122	0.02839969	0.02661355
OTU_11 (unclassified)	0.00000000	0.00000000	0.03604603	0.01279146	0.00784040	0.01466135
OTU_12 (unclassified)	0.03355381	0.00000000	0.00000000	0.00080403	0.00000000	0.00039841
OTU_13 (Asticcacaulis)	0.00089569	0.00773301	0.00908083	0.00745560	0.00818887	0.00940239
OTU_14 (unclassified)	0.00013780	0.01912902	0.00561486	0.01096411	0.00818887	0.01011952
OTU_15 (unclassified)	0.00268706	0.00398860	0.00845695	0.00796725	0.00845021	0.00701195
OTU_16 (Streptophyta)	0.00020670	0.01066341	0.00866491	0.00665156	0.00958272	0.00828685
OTU_17 (Geobacillus)	0.00613201	0.01538462	0.01254679	0.01534976	0.00958272	0.01498008
OTU_18 (Cryobacterium)	0.00062009	0.02246642	0.01434909	0.01227980	0.01045387	0.02318725
OTU_19 (unclassified)	0.02418355	0.00000000	0.00000000	0.00014619	0.00008712	0.00039841
OTU_20 (unclassified)	0.01398650	0.00000000	0.00201026	0.00051166	0.00087116	0.00167331
OTU_21 (Lactobacillus)	0.00875017	0.02612943	0.03479828	0.01578832	0.01367715	0.01593625
OTU_22 (Propionibacterium)	0.00089569	0.00602361	0.01233883	0.00416636	0.00453001	0.00677291
OTU_23 (unclassified)	0.00000000	0.00000000	0.02065715	0.00621300	0.00174231	0.00988048
OTU_24 (Tetragenococcus)	0.00757889	0.03606024	0.02849023	0.01315693	0.00810175	0.00948207
OTU_25 (unclassified)	0.01260852	0.00000000	0.00000000	0.00043856	0.00000000	0.00000000
OTU_26 (Pseudomonas)	0.00124018	0.00398860	0.00443643	0.00285067	0.00139385	0.00207171
OTU_27 (unclassified)	0.01694915	0.00000000	0.00000000	0.00021928	0.00000000	0.00000000
OTU_28 (Acidovorax)	0.00013780	0.01579162	0.00956606	0.00862510	0.00592386	0.00772908
OTU_29 (Curvibacter)	0.00027560	0.01286121	0.00616942	0.00555515	0.00444290	0.00621514
OTU_30 (Ralstonia)	0.00137798	0.00724461	0.00561486	0.00380089	0.00383309	0.00462151
OTU_31 (Sphingomonas)	0.00027560	0.01196581	0.00686261	0.01016008	0.00566251	0.00741036
OTU_32	0.00165358	0.00740741	0.00436711	0.00613990	0.00278770	0.00525896
OTU_33 (Flavobacterium)	0.00000000	0.00960521	0.00873423	0.00445874	0.00331039	0.00358566
OTU_34 (TM7_genus_incertae_sedis)	0.00813008	0.00000000	0.00000000	0.00043856	0.00000000	0.00015936
OTU_35 (Rhodobacter)	0.00916357	0.00024420	0.00000000	0.00007309	0.00000000	0.00000000
OTU_36 (Serratia)	0.00117128	0.01188441	0.00256481	0.00482421	0.00548828	0.00525896
OTU_37 (unclassified)	0.01391760	0.00000000	0.00000000	0.00058475	0.00000000	0.00000000
OTU_38 (unclassified)	0.00985256	0.00000000	0.00000000	0.00138879	0.00008712	0.00000000
OTU_39 (unclassified)	0.00006890	0.00830281	0.00325801	0.00365470	0.00278770	0.00294821
OTU_40 (TM7_genus_incertae_sedis)	0.00000000	0.00757021	0.00000000	0.00000000	0.00034846	0.00000000
OTU_41 (unclassified)	0.00027560	0.00529101	0.00180230	0.00168116	0.00270058	0.00135458
OTU_42 (Spirosoma)	0.00000000	0.00748881	0.00547622	0.00306995	0.00278770	0.00334661
OTU_43 (unclassified)	0.00013780	0.00008140	0.01774574	0.00087713	0.00069692	0.00278884
OTU_44 (Methylobacterium)	0.00055119	0.00618641	0.00817968	0.00628609	0.00627232	0.01035857
OTU_45 (unclassified)	0.01446879	0.00008140	0.00000000	0.00065785	0.00034846	0.00000000
OTU_46 (Methylobacterium)	0.00000000	0.00480260	0.00124775	0.00095022	0.00026135	0.00127490
OTU_47 (unclassified)	0.00006890	0.00919821	0.00263413	0.00204663	0.00235212	0.00223108
OTU_48 (unclassified)	0.00000000	0.00284900	0.00388188	0.00175426	0.00148097	0.00199203

OTU_49 (Aeribacillus)	0.00103348	0.00748881	0.00395120	0.01111030	0.00827598	0.00653386
OTU_50 (Lactobacillus)	0.00103348	0.00618641	0.00311937	0.00160807	0.00209077	0.00095618
OTU_51 (GpXI)	0.00000000	0.00317460	0.00173298	0.00095022	0.00026135	0.00087649
OTU_52 (Meiothermus)	0.00020670	0.00284900	0.00166366	0.00168116	0.00130673	0.00159363
OTU_53 (unclassified)	0.00000000	0.00000000	0.00436711	0.00109641	0.00139385	0.00326693
OTU_54 (Methylobacterium)	0.00020670	0.00130240	0.00159434	0.00087713	0.00043558	0.00111554
OTU_55 (unclassified)	0.00000000	0.00529101	0.00173298	0.00219282	0.00121962	0.00063745
OTU_56 (Ilumatobacter)	0.01095494	0.00000000	0.00000000	0.00029238	0.00000000	0.00007968
OTU_57 (unclassified)	0.00489183	0.00000000	0.00000000	0.00029238	0.00339751	0.00000000
OTU_58 (unclassified)	0.00000000	0.00757021	0.00415916	0.00277757	0.00243924	0.00167331
OTU_59 (Ilumatobacter)	0.00523632	0.00056980	0.00027728	0.00000000	0.00008712	0.00000000
OTU_60 (OD1_genus_incertae_sedis)	0.00000000	0.00602361	0.00263413	0.00387399	0.00139385	0.00135458
OTU_61 (Cupriavidus)	0.00068899	0.00309320	0.00270345	0.00328923	0.00130673	0.00183267
OTU_62 (unclassified)	0.00062009	0.00358160	0.00138639	0.00109641	0.00130673	0.00063745
OTU_63 (Flavobacterium)	0.00392724	0.00000000	0.00000000	0.00000000	0.00000000	0.00000000
OTU_64 (Lactobacillus)	0.00000000	0.00000000	0.00000000	0.01293765	0.04338357	0.00000000
OTU_65 (Microbacterium)	0.00013780	0.00504681	0.00305005	0.00138879	0.00034846	0.00294821
OTU_66 (unclassified)	0.00000000	0.00423280	0.00173298	0.00058475	0.00104539	0.00063745
OTU_67 (unclassified)	0.01522668	0.00000000	0.00000000	0.00058475	0.00000000	0.00000000
OTU_68 (unclassified)	0.00000000	0.00529101	0.00180230	0.00175426	0.00087116	0.00095618
OTU_69 (Polynucleobacter)	0.00020670	0.00480260	0.00478303	0.00241210	0.00226501	0.00438247
OTU_70 (Aeromonas)	0.00020670	0.00000000	0.02058783	0.03640085	0.03153585	0.01155378
OTU_71 (Caldicellulosiruptor)	0.00075789	0.00219780	0.00547622	0.00416636	0.00235212	0.00509960
OTU_72 (unclassified)	0.00020670	0.00667481	0.00402052	0.00175426	0.00104539	0.00143426
OTU_73 (unclassified)	0.00399614	0.00000000	0.00000000	0.00036547	0.00017423	0.00000000
OTU_74 (unclassified)	0.00516742	0.00032560	0.00000000	0.00043856	0.00000000	0.00015936
OTU_75 (unclassified)	0.00626981	0.00000000	0.00000000	0.00000000	0.00000000	0.00007968
OTU_76 (unclassified)	0.00013780	0.00284900	0.00187162	0.00021928	0.00000000	0.00095618
OTU_77 (Acinetobacter)	0.00013780	0.00284900	0.00277277	0.00160807	0.00156808	0.00119522
OTU_78 (Paracoccus)	0.02197878	0.00000000	0.00000000	0.00087713	0.00008712	0.00071713
OTU_79 (unclassified)	0.00496073	0.00000000	0.00000000	0.00000000	0.00000000	0.00000000
OTU_80 (unclassified)	0.00303156	0.00000000	0.00000000	0.00000000	0.00000000	0.00000000
OTU_81 (Bdellovibrio)	0.00000000	0.00227920	0.00228754	0.00051166	0.00095827	0.00127490
OTU_82 (unclassified)	0.00117128	0.00366300	0.00083183	0.00124260	0.00000000	0.00007968
OTU_83 (unclassified)	0.00006890	0.00236060	0.00187162	0.00248520	0.00121962	0.00159363
OTU_84 (unclassified)	0.00000000	0.00130240	0.00194094	0.00021928	0.00034846	0.00079681
OTU_85 (Burkholderia)	0.00034449	0.00179080	0.00117843	0.00095022	0.00174231	0.00111554
OTU_86 (unclassified)	0.00000000	0.00219780	0.00187162	0.00095022	0.00104539	0.00119522
OTU_87 (unclassified)	0.00006890	0.00227920	0.00187162	0.00146188	0.00052269	0.00079681
OTU_88 (Ilumatobacter)	0.00130908	0.00097680	0.00076251	0.00029238	0.00034846	0.00031873
OTU_89 (unclassified)	0.00716550	0.00056980	0.00034660	0.00080403	0.00026135	0.00039841
OTU_90 (Flavobacterium)	0.00000000	0.00000000	0.00436711	0.00051166	0.00060981	0.00143426
OTU_91 (Anoxybacillus)	0.00137798	0.00227920	0.00090115	0.00087713	0.00130673	0.00135458
OTU_92 (unclassified)	0.00227367	0.00048840	0.00000000	0.00021928	0.00008712	0.00000000
OTU_93 (Bacillus)	0.00303156	0.00431420	0.00492167	0.00285067	0.00322328	0.00446215
OTU_94 (unclassified)	0.00723439	0.00000000	0.00000000	0.00036547	0.00000000	0.00000000
OTU_95 (Chryseobacterium)	0.00013780	0.00398860	0.00187162	0.00080403	0.00043558	0.00047809
OTU_96 (unclassified)	0.00737219	0.00056980	0.00034660	0.00014619	0.00008712	0.00015936
OTU_97 (Acinetobacter)	0.00296266	0.00870981	0.00769444	0.00248520	0.00243924	0.00302789
OTU_98 (unclassified)	0.00675210	0.00000000	0.00000000	0.00014619	0.00008712	0.00000000

OTU_99 (unclassified)	0.00358275	0.00195360	0.00027728	0.00000000	0.00000000	0.00000000
OTU_100 (TM7_genus_incertae_sedis)	0.00282486	0.00000000	0.00000000	0.00000000	0.00000000	0.00000000
OTU_101 (Ilumatobacter)	0.00509853	0.00000000	0.00000000	0.00000000	0.00000000	0.00000000
OTU_102 (Zimmermannella)	0.00041339	0.00105820	0.00270345	0.00197354	0.00165520	0.00223108
OTU_103 (Cetobacterium)	0.00537412	0.00000000	0.00000000	0.00058475	0.00008712	0.00007968
OTU_104 (unclassified)	0.00399614	0.00000000	0.00000000	0.00007309	0.00008712	0.00015936
OTU_105 (Legionella)	0.00365165	0.00065120	0.00000000	0.00007309	0.00000000	0.00000000
OTU_106 (TM7_genus_incertae_sedis)	0.00000000	0.00219780	0.00117843	0.00058475	0.00043558	0.00047809
OTU_107 (unclassified)	0.00000000	0.00260480	0.00027728	0.00021928	0.00043558	0.00047809
OTU_108 (Shewanella)	0.00620091	0.00000000	0.00000000	0.00314305	0.00766617	0.00031873
OTU_109 (Chryseobacterium)	0.00000000	0.00252340	0.00076251	0.00073094	0.00034846	0.00031873
OTU_110 (TM7_genus_incertae_sedis)	0.00000000	0.00016280	0.00207958	0.00073094	0.00087116	0.00071713
OTU_111 (Microcella)	0.00289376	0.00016280	0.00000000	0.00000000	0.00008712	0.00000000
OTU_112 (Rhizobium)	0.00778559	0.00000000	0.00000000	0.00000000	0.00000000	0.00000000
OTU_113 (unclassified)	0.00000000	0.00179080	0.00041592	0.00000000	0.00008712	0.00000000
OTU_114 (unclassified)	0.00000000	0.00122100	0.00103979	0.00007309	0.00026135	0.00047809
OTU_115 (unclassified)	0.00000000	0.00122100	0.00048523	0.00043856	0.00017423	0.00015936
OTU_116 (Lactobacillus)	0.00124018	0.00781441	0.00429780	0.00358161	0.00130673	0.00135458
OTU_117 (unclassified)	0.00316935	0.00000000	0.00000000	0.00000000	0.00000000	0.00000000
OTU_118 (unclassified)	0.00275596	0.00000000	0.00000000	0.00014619	0.00000000	0.00000000
OTU_119 (unclassified)	0.00254926	0.00000000	0.00000000	0.00000000	0.00000000	0.00000000
OTU_120 (unclassified)	0.00310045	0.00000000	0.00000000	0.00000000	0.00000000	0.00007968
OTU_121 (unclassified)	0.00806118	0.00008140	0.00000000	0.00000000	0.00000000	0.00007968
OTU_122 (Geobacillus)	0.00068899	0.00089540	0.00256481	0.00131569	0.00139385	0.00262948
OTU_123 (unclassified)	0.00041339	0.00065120	0.00069319	0.00021928	0.00060981	0.00000000
OTU_124 (unclassified)	0.00303156	0.00008140	0.00000000	0.00000000	0.00000000	0.00000000
OTU_125 (Pelomonas)	0.00000000	0.00358160	0.00117843	0.00138879	0.00052269	0.00119522
OTU_126 (Thermus)	0.00013780	0.00586081	0.00187162	0.02002778	0.01437407	0.02000000
OTU_127 (Ilumatobacter)	0.00248036	0.00000000	0.00000000	0.00000000	0.00000000	0.00000000
OTU_128 (unclassified)	0.00000000	0.00293040	0.00097047	0.00036547	0.00000000	0.00023904
OTU_129 (Flavobacterium)	0.00000000	0.00236060	0.00048523	0.00007309	0.00000000	0.00023904
OTU_130 (unclassified)	0.00144688	0.00000000	0.00000000	0.00007309	0.00000000	0.00000000
OTU_131 (unclassified)	0.00151578	0.00000000	0.00000000	0.00058475	0.00000000	0.00015936
OTU_132 (unclassified)	0.00062009	0.00301180	0.00110911	0.00409327	0.00522694	0.00446215
OTU_133 (Deinococcus)	0.00068899	0.00260480	0.00214890	0.00102332	0.00182943	0.00247012
OTU_134 (unclassified)	0.00372055	0.00000000	0.00000000	0.00000000	0.00008712	0.00000000
OTU_135 (Rhodococcus)	0.00006890	0.00105820	0.00062387	0.00087713	0.00026135	0.00023904
OTU_136 (Arcicella)	0.00006890	0.00081400	0.00110911	0.00182735	0.00052269	0.00055777
OTU_137 (Burkholderia)	0.00055119	0.00195360	0.00228754	0.00248520	0.00374597	0.00342629
OTU_138 (unclassified)	0.00000000	0.00105820	0.00076251	0.00014619	0.00000000	0.00007968
OTU_139 (Thermus)	0.00013780	0.00138380	0.00159434	0.00007309	0.00034846	0.00143426
OTU_140 (Micrococcus)	0.00020670	0.00040700	0.00117843	0.00029238	0.00078404	0.00119522
OTU_141 (unclassified)	0.00000000	0.00073260	0.00034660	0.00029238	0.00043558	0.00007968
OTU_142 (unclassified)	0.00034449	0.00073260	0.00027728	0.00102332	0.00008712	0.00000000
OTU_143 (OD1_genus_incertae_sedis)	0.00000000	0.00195360	0.00041592	0.00116951	0.00087116	0.00023904
OTU_144 (Sphingomonas)	0.00027560	0.00000000	0.00076251	0.00007309	0.00017423	0.00015936
OTU_145 (unclassified)	0.00000000	0.00293040	0.00048523	0.00036547	0.00000000	0.00023904

OTU_146 (unclassified)	0.00027560	0.00203500	0.00055455	0.00058475	0.00008712	0.00023904
OTU_147 (Lactobacillus)	0.00062009	0.00268620	0.00318869	0.00182735	0.00043558	0.00135458
OTU_148 (Bacillariophyta)	0.00000000	0.00341880	0.00173298	0.00058475	0.00052269	0.00055777
OTU_149 (unclassified)	0.00000000	0.00089540	0.00083183	0.00021928	0.00017423	0.00031873
OTU_150 (unclassified)	0.00000000	0.00162800	0.00062387	0.00036547	0.00043558	0.00039841
OTU_151 (Rhodobacter)	0.00833678	0.00024420	0.00020796	0.00065785	0.00000000	0.00007968
OTU_152 (Bradyrhizobium)	0.00192917	0.00390720	0.00298073	0.00336233	0.00113250	0.00239044
OTU_153 (unclassified)	0.00000000	0.00056980	0.00041592	0.00000000	0.00000000	0.00007968
OTU_154 (Flavobacterium)	0.00020670	0.00113960	0.00041592	0.00007309	0.00008712	0.00000000
OTU_155 (unclassified)	0.00144688	0.00016280	0.00000000	0.00000000	0.00026135	0.00000000
OTU_156 (unclassified)	0.00027560	0.00056980	0.00090115	0.00029238	0.00017423	0.00023904
OTU_157 (unclassified)	0.00000000	0.00024420	0.00117843	0.00058475	0.00026135	0.00087649
OTU_158 (unclassified)	0.00000000	0.00170940	0.00076251	0.00124260	0.00113250	0.00055777
OTU_159 (unclassified)	0.00000000	0.00350020	0.00090115	0.00095022	0.00078404	0.00071713
OTU_160 (unclassified)	0.00213587	0.00000000	0.00000000	0.00000000	0.00000000	0.00000000
OTU_161 (Novosphingobium)	0.00027560	0.00105820	0.00034660	0.00073094	0.00043558	0.00023904
OTU_162 (Methylotenera)	0.00000000	0.00472120	0.00138639	0.00073094	0.00069692	0.00055777
OTU_163 (Microbacterium)	0.00117128	0.00000000	0.00000000	0.00000000	0.00000000	0.00000000
OTU_164 (unclassified)	0.00000000	0.00000000	0.00103979	0.00000000	0.00000000	0.00023904
OTU_165 (unclassified)	0.00013780	0.00040700	0.00062387	0.00000000	0.00034846	0.00007968
OTU_166 (unclassified)	0.00165358	0.00000000	0.00000000	0.00000000	0.00000000	0.00000000
OTU_167 (Arthrobacter)	0.00000000	0.00146520	0.00062387	0.00095022	0.00043558	0.00079681
OTU_168 (unclassified)	0.00165358	0.00000000	0.00000000	0.00014619	0.00000000	0.00000000
OTU_169 (unclassified)	0.00241146	0.00000000	0.00000000	0.00000000	0.00000000	0.00007968
OTU_170 (Roseomonas)	0.00165358	0.00000000	0.00000000	0.00000000	0.00000000	0.00000000
OTU_171 (Corynebacterium)	0.00048229	0.00000000	0.00124775	0.00131569	0.00217789	0.00063745
OTU_172 (TM7_genus_incertae_sedis)	0.00000000	0.00089540	0.00020796	0.00051166	0.00008712	0.00007968
OTU_173 (TM7_genus_incertae_sedis)	0.00096459	0.00000000	0.00000000	0.00000000	0.00000000	0.00000000
OTU_174 (Clostridium_XI)	0.00172247	0.00000000	0.00000000	0.00000000	0.00000000	0.00000000
OTU_175 (unclassified)	0.00000000	0.00195360	0.00041592	0.00095022	0.00052269	0.00159363
OTU_176 (Microbacterium)	0.00034449	0.00024420	0.00062387	0.00007309	0.00017423	0.00047809
OTU_177 (unclassified)	0.00006890	0.00146520	0.00000000	0.00000000	0.00008712	0.00000000
OTU_178 (unclassified)	0.00006890	0.00000000	0.00055455	0.00000000	0.00000000	0.00007968
OTU_179 (unclassified)	0.00000000	0.00000000	0.00062387	0.00007309	0.00026135	0.00023904
OTU_180 (unclassified)	0.00427174	0.00008140	0.00000000	0.00007309	0.00000000	0.00000000
OTU_181 (TM7_genus_incertae_sedis)	0.00000000	0.00000000	0.00152502	0.00000000	0.00000000	0.00000000
OTU_182 (unclassified)	0.00124018	0.00000000	0.00000000	0.00000000	0.00000000	0.00000000
OTU_183 (Sphingomonas)	0.00000000	0.00000000	0.00000000	0.00116951	0.00095827	0.00215139
OTU_184 (unclassified)	0.00110238	0.00000000	0.00000000	0.00000000	0.00000000	0.00000000
OTU_185 (OD1_genus_incertae_sedis)	0.00000000	0.00154660	0.00020796	0.00000000	0.00017423	0.00031873
OTU_186 (TM7_genus_incertae_sedis)	0.00020670	0.00073260	0.00027728	0.00000000	0.00008712	0.00007968
OTU_187 (unclassified)	0.00020670	0.00122100	0.00000000	0.00065785	0.00078404	0.00031873
OTU_188 (Nubsella)	0.00000000	0.00154660	0.00069319	0.00036547	0.00026135	0.00031873
OTU_189 (Roseococcus)	0.00248036	0.00000000	0.00000000	0.00000000	0.00000000	0.00000000
OTU_190 (Bacillus)	0.00062009	0.00227920	0.00180230	0.00109641	0.00060981	0.00111554
OTU_191 (unclassified)	0.00172247	0.00016280	0.00083183	0.00029238	0.00017423	0.00047809

OTU_192 (TM7_genus_incertae_sedis)	0.00000000	0.00105820	0.00083183	0.00007309	0.00017423	0.00015936
OTU_193 (unclassified)	0.00206697	0.00000000	0.00000000	0.00021928	0.00174231	0.00071713
OTU_194 (Caldicellulosiruptor)	0.00041339	0.00065120	0.00131707	0.00116951	0.00139385	0.00175299
OTU_195 (SR1_genus_incertae_sedis)	0.00041339	0.00097680	0.00076251	0.00007309	0.00000000	0.00039841
OTU_196 (unclassified)	0.00220477	0.00016280	0.00006932	0.00007309	0.00000000	0.00000000
OTU_197 (Erythrobacter)	0.00000000	0.00016280	0.00062387	0.00036547	0.00008712	0.00031873
OTU_198 (unclassified)	0.00055119	0.00081400	0.00076251	0.00000000	0.00000000	0.00007968
OTU_199 (unclassified)	0.00199807	0.00000000	0.00000000	0.00000000	0.00000000	0.00000000
OTU_200 (Rudaea)	0.00000000	0.00000000	0.00090115	0.00000000	0.00017423	0.00023904
OTU_201 (unclassified)	0.00000000	0.00065120	0.00041592	0.00007309	0.00000000	0.00000000
OTU_202 (unclassified)	0.00220477	0.00000000	0.00000000	0.00000000	0.00008712	0.00000000
OTU_203 (unclassified)	0.00124018	0.00008140	0.00000000	0.00000000	0.00000000	0.00000000
OTU_204 (unclassified)	0.00186027	0.00000000	0.00000000	0.00029238	0.00000000	0.00000000
OTU_205 (unclassified)	0.00000000	0.00227920	0.00027728	0.00021928	0.00026135	0.00039841
OTU_206 (unclassified)	0.00110238	0.00000000	0.00000000	0.00000000	0.00000000	0.00000000
OTU_207 (unclassified)	0.00000000	0.00105820	0.00013864	0.00051166	0.00017423	0.00015936
OTU_208 (unclassified)	0.00000000	0.00325600	0.00194094	0.00051166	0.00026135	0.00031873
OTU_209 (unclassified)	0.00082679	0.00000000	0.00000000	0.00014619	0.00000000	0.00000000
OTU_210 (Janthinobacterium)	0.00000000	0.00097680	0.00083183	0.00095022	0.00008712	0.00111554
OTU_211 (Sphingobium)	0.00000000	0.00146520	0.00000000	0.00007309	0.00017423	0.00087649
OTU_212 (Comamonas)	0.00000000	0.00113960	0.00069319	0.00029238	0.00000000	0.00023904
OTU_213 (Ilumatobacter)	0.00206697	0.00016280	0.00000000	0.00000000	0.00000000	0.00023904
OTU_214 (unclassified)	0.00158468	0.00000000	0.00000000	0.00000000	0.00000000	0.00000000
OTU_215 (Bacillus)	0.00158468	0.00309320	0.00228754	0.00160807	0.00121962	0.00095618
OTU_216 (Hyphomicrobium)	0.00110238	0.00000000	0.00000000	0.00000000	0.00000000	0.00000000
OTU_217 (Staphylococcus)	0.00006890	0.00073260	0.00166366	0.00014619	0.00008712	0.00047809
OTU_218 (Sphingomonas)	0.00034449	0.00170940	0.00097047	0.00000000	0.00052269	0.00007968
OTU_219 (Acinetobacter)	0.00000000	0.00463980	0.00103979	0.00204663	0.00130673	0.00127490
OTU_220 (unclassified)	0.00000000	0.00097680	0.00069319	0.00007309	0.00000000	0.00000000
OTU_221 (Bdellovibrio)	0.00000000	0.00130240	0.00090115	0.00029238	0.00000000	0.00015936
OTU_222 (Neisseria)	0.00000000	0.00065120	0.00041592	0.00073094	0.00026135	0.00039841
OTU_223 (Roseomonas)	0.00137798	0.00000000	0.00000000	0.00000000	0.00000000	0.00000000
OTU_224 (Mycobacterium)	0.00006890	0.00008140	0.00069319	0.00000000	0.00043558	0.00047809
OTU_225 (TM7_genus_incertae_sedis)	0.00000000	0.00016280	0.00055455	0.00007309	0.00000000	0.00007968
OTU_226 (Sphingomonas)	0.00000000	0.00000000	0.00000000	0.00021928	0.00000000	0.00063745
OTU_227 (Flavobacterium)	0.00000000	0.00081400	0.00041592	0.00000000	0.00052269	0.00055777
OTU_228 (unclassified)	0.00137798	0.00000000	0.00000000	0.00000000	0.00000000	0.00000000
OTU_229 (Streptococcus)	0.00020670	0.00024420	0.00187162	0.00000000	0.00017423	0.00047809
OTU_230 (unclassified)	0.00110238	0.00040700	0.00020796	0.00007309	0.00017423	0.00015936
OTU_231 (unclassified)	0.00000000	0.00000000	0.00103979	0.00007309	0.00008712	0.00007968
OTU_232 (unclassified)	0.00055119	0.00032560	0.00027728	0.00000000	0.00000000	0.00000000
OTU_233 (GpIIa)	0.00000000	0.00097680	0.00006932	0.00007309	0.00000000	0.00007968
OTU_234 (Elizabethkingia)	0.00000000	0.00040700	0.00069319	0.00021928	0.00000000	0.00039841
OTU_235 (Legionella)	0.00227367	0.00000000	0.00000000	0.00000000	0.00000000	0.00000000
OTU_236 (unclassified)	0.00000000	0.00113960	0.00000000	0.00000000	0.00000000	0.00000000
OTU_237 (unclassified)	0.00000000	0.00056980	0.00006932	0.00000000	0.00008712	0.00007968
OTU_238 (Hydrogenophaga)	0.00000000	0.00382580	0.00055455	0.00124260	0.00148097	0.00223108
OTU_239 (unclassified)	0.00000000	0.00179080	0.00027728	0.00043856	0.00026135	0.00031873

OTU_240 (OD1_genus_incertae_sedis)	0.00000000	0.00016280	0.00076251	0.00007309	0.00034846	0.00000000
OTU_241 (unclassified)	0.00179137	0.00000000	0.00000000	0.00000000	0.00008712	0.00000000
OTU_242 (unclassified)	0.00000000	0.00130240	0.00090115	0.00065785	0.00043558	0.00047809
OTU_243 (Leifsonia)	0.00075789	0.00000000	0.00000000	0.00000000	0.00000000	0.00000000
OTU_244 (Erythromicrobium)	0.00000000	0.00016280	0.00076251	0.00007309	0.00017423	0.00039841
OTU_245 (unclassified)	0.00075789	0.00000000	0.00000000	0.00000000	0.00000000	0.00000000
OTU_246 (Enhydrobacter)	0.00000000	0.00056980	0.00270345	0.00065785	0.00026135	0.00111554
OTU_247 (Microbacterium)	0.00006890	0.00113960	0.00069319	0.00073094	0.00060981	0.00055777
OTU_248 (unclassified)	0.00020670	0.00097680	0.00055455	0.00043856	0.00060981	0.00047809
OTU_249 (Gp4)	0.00000000	0.00113960	0.00076251	0.00080403	0.00069692	0.00087649
OTU_250 (unclassified)	0.00006890	0.00105820	0.00006932	0.00029238	0.00008712	0.00023904
OTU_251 (unclassified)	0.00000000	0.00000000	0.00048523	0.00000000	0.00000000	0.00007968
OTU_252 (unclassified)	0.00055119	0.00000000	0.00000000	0.00000000	0.00000000	0.00000000
OTU_253 (unclassified)	0.00000000	0.00065120	0.00013864	0.00029238	0.00000000	0.00000000
OTU_254 (Methylobacterium)	0.00000000	0.00056980	0.00000000	0.00051166	0.00008712	0.00000000
OTU_255 (Phenylobacterium)	0.00000000	0.00065120	0.00020796	0.00051166	0.00017423	0.00031873
OTU_256 (Eikenella)	0.00000000	0.00000000	0.00055455	0.00000000	0.00008712	0.00015936
OTU_257 (unclassified)	0.00006890	0.00048840	0.00013864	0.00029238	0.00000000	0.00000000
OTU_258 (unclassified)	0.00000000	0.00146520	0.00041592	0.00036547	0.00069692	0.00031873
OTU_259 (Ilumatobacter)	0.00089569	0.00000000	0.00000000	0.00029238	0.00000000	0.00000000
OTU_260 (unclassified)	0.00089569	0.00008140	0.00006932	0.00021928	0.00000000	0.00000000
OTU_261 (unclassified)	0.00000000	0.00154660	0.00062387	0.00029238	0.00017423	0.00039841
OTU_262 (TM7_genus_incertae_sedis)	0.00000000	0.00170940	0.00048523	0.00000000	0.00008712	0.00007968
OTU_263 (Sphingomonas)	0.00000000	0.00000000	0.00000000	0.00168116	0.00139385	0.00207171
OTU_264 (Brevibacillus)	0.00000000	0.00016280	0.00048523	0.00752869	0.00792752	0.01378486
OTU_265 (unclassified)	0.00000000	0.00154660	0.00013864	0.00124260	0.00060981	0.00015936
OTU_266 (unclassified)	0.00124018	0.00000000	0.00000000	0.00007309	0.00000000	0.00000000
OTU_267 (unclassified)	0.00000000	0.00000000	0.00041592	0.00000000	0.00000000	0.00000000
OTU_268 (Roseomonas)	0.00144688	0.00000000	0.00000000	0.00000000	0.00000000	0.00000000
OTU_269 (Marmoricola)	0.00000000	0.00008140	0.00062387	0.00014619	0.00000000	0.00031873
OTU_270 (unclassified)	0.00213587	0.00000000	0.00000000	0.00000000	0.00000000	0.00000000
OTU_271 (Sphingomonas)	0.00000000	0.00000000	0.00000000	0.00029238	0.00121962	0.00231076
OTU_272 (Lactobacillus)	0.00000000	0.00000000	0.00000000	0.04941159	0.01472254	0.00000000
OTU_273 (unclassified)	0.00048229	0.00000000	0.00000000	0.00000000	0.00000000	0.00000000
OTU_274 (unclassified)	0.00013780	0.00073260	0.00034660	0.00014619	0.00043558	0.00023904
OTU_275 (unclassified)	0.00000000	0.00081400	0.00069319	0.00007309	0.00000000	0.00007968
OTU_276 (unclassified)	0.00227367	0.00000000	0.00000000	0.00000000	0.00000000	0.00000000
OTU_277 (unclassified)	0.00006890	0.00040700	0.00076251	0.00029238	0.00034846	0.00055777
OTU_278 (TM7_genus_incertae_sedis)	0.00000000	0.00000000	0.00048523	0.00000000	0.00000000	0.00000000
OTU_279 (unclassified)	0.00000000	0.00073260	0.00000000	0.00000000	0.00008712	0.00000000
OTU_280 (unclassified)	0.00000000	0.00000000	0.00152502	0.00058475	0.00060981	0.00023904
OTU_281 (Rheinheimera)	0.00006890	0.00081400	0.00055455	0.00007309	0.00017423	0.00055777
OTU_282 (unclassified)	0.00130908	0.00000000	0.00000000	0.00000000	0.00000000	0.00000000
OTU_283 (unclassified)	0.00075789	0.00000000	0.00000000	0.00000000	0.00000000	0.00000000
OTU_284 (unclassified)	0.00000000	0.00097680	0.00000000	0.00087713	0.00008712	0.00007968
OTU_285 (unclassified)	0.00006890	0.00130240	0.00006932	0.00043856	0.00043558	0.00015936
OTU_286 (unclassified)	0.00000000	0.00000000	0.00110911	0.00000000	0.00000000	0.00023904
OTU_287 (unclassified)	0.00000000	0.00065120	0.00013864	0.00000000	0.00000000	0.00000000

OTU_288 (unclassified)	0.00000000	0.00179080	0.00027728	0.00080403	0.00043558	0.00031873
OTU_289 (Armatimonas_Armatimonadetes_gp1)	0.00000000	0.00073260	0.00055455	0.00021928	0.00000000	0.00031873
OTU_290 (unclassified)	0.00000000	0.00000000	0.00103979	0.00014619	0.00008712	0.00023904
OTU_291 (unclassified)	0.00000000	0.00000000	0.00048523	0.00000000	0.00000000	0.00015936
OTU_292 (OD1_genus_incertae_sedis)	0.00000000	0.00081400	0.00000000	0.00000000	0.00000000	0.00000000
OTU_293 (Dermacoccus)	0.00020670	0.00162800	0.00006932	0.00021928	0.00034846	0.00023904
OTU_294 (Brevibacillus)	0.00000000	0.00000000	0.00006932	0.00095022	0.00095827	0.00358566
OTU_295 (Comamonas)	0.00000000	0.00056980	0.00027728	0.00000000	0.00000000	0.00000000
OTU_296 (unclassified)	0.00000000	0.00211640	0.00117843	0.00007309	0.00069692	0.00047809
OTU_297 (Legionella)	0.00013780	0.00113960	0.00020796	0.00021928	0.00008712	0.00000000
OTU_298 (unclassified)	0.00000000	0.00040700	0.00000000	0.00000000	0.00000000	0.00000000
OTU_299 (Iamia)	0.00096459	0.00000000	0.00000000	0.00000000	0.00000000	0.00000000
OTU_300 (Flectobacillus)	0.00000000	0.00073260	0.00041592	0.00036547	0.00000000	0.00000000
OTU_301 (unclassified)	0.00055119	0.00000000	0.00013864	0.00000000	0.00000000	0.00000000
OTU_302 (unclassified)	0.00000000	0.00048840	0.00000000	0.00000000	0.00000000	0.00000000
OTU_303 (unclassified)	0.00041339	0.00000000	0.00000000	0.00000000	0.00000000	0.00000000
OTU_304 (Mesorhizobium)	0.00020670	0.00138380	0.00062387	0.00095022	0.00078404	0.00151394
OTU_305 (unclassified)	0.00000000	0.00000000	0.00069319	0.00051166	0.00008712	0.00015936
OTU_306 (unclassified)	0.00006890	0.00032560	0.00069319	0.00029238	0.00078404	0.00095618
OTU_307 (unclassified)	0.00000000	0.00056980	0.00000000	0.00000000	0.00000000	0.00000000
OTU_308 (unclassified)	0.00000000	0.00146520	0.00097047	0.00014619	0.00026135	0.00023904
OTU_309 (unclassified)	0.00172247	0.00000000	0.00000000	0.00007309	0.00000000	0.00000000
OTU_310 (Arthrobacter)	0.00000000	0.00187220	0.00117843	0.00036547	0.00000000	0.00055777
OTU_311 (unclassified)	0.00000000	0.00073260	0.00034660	0.00043856	0.00000000	0.00031873
OTU_312 (Lactococcus)	0.00000000	0.00000000	0.00000000	0.00540896	0.00845021	0.00000000
OTU_313 (Pseudomonas)	0.00006890	0.00024420	0.00027728	0.00000000	0.00008712	0.00000000
OTU_314 (unclassified)	0.00048229	0.00089540	0.00006932	0.00000000	0.00000000	0.00000000
OTU_315 (Sphingopyxis)	0.00000000	0.00032560	0.00034660	0.00000000	0.00000000	0.00031873
OTU_316 (unclassified)	0.00000000	0.00040700	0.00013864	0.00043856	0.00008712	0.00000000
OTU_317 (Limnohabitans)	0.00013780	0.00146520	0.00180230	0.00051166	0.00008712	0.00031873
OTU_318 (unclassified)	0.00000000	0.00048840	0.00000000	0.00007309	0.00000000	0.00000000
OTU_319 (unclassified)	0.00151578	0.00000000	0.00000000	0.00000000	0.00000000	0.00000000
OTU_320 (unclassified)	0.00151578	0.00000000	0.00000000	0.00000000	0.00000000	0.00000000
OTU_321 (unclassified)	0.00096459	0.00000000	0.00000000	0.00000000	0.00000000	0.00000000
OTU_322 (unclassified)	0.00006890	0.00048840	0.00020796	0.00065785	0.00034846	0.00007968
OTU_323 (unclassified)	0.00213587	0.00000000	0.00034660	0.00007309	0.00000000	0.00007968
OTU_324 (unclassified)	0.00000000	0.00016280	0.00055455	0.00007309	0.00008712	0.00007968
OTU_325 (unclassified)	0.00248036	0.00000000	0.00000000	0.00014619	0.00000000	0.00000000
OTU_326 (Petrobacter)	0.00027560	0.00008140	0.00041592	0.00029238	0.00000000	0.00023904
OTU_327 (Chitinibacter)	0.00048229	0.00008140	0.00034660	0.00160807	0.00261347	0.00167331
OTU_328 (unclassified)	0.00000000	0.00040700	0.00000000	0.00000000	0.00000000	0.00000000
OTU_329 (unclassified)	0.00034449	0.00000000	0.00000000	0.00000000	0.00000000	0.00000000
OTU_330 (unclassified)	0.00000000	0.00105820	0.00006932	0.00000000	0.00026135	0.00000000
OTU_331	0.00006890	0.00170940	0.00069319	0.00073094	0.00052269	0.00071713
OTU_332 (unclassified)	0.00000000	0.00000000	0.00034660	0.00014619	0.00026135	0.00000000
OTU_333 (unclassified)	0.00000000	0.00048840	0.00000000	0.00000000	0.00000000	0.00000000
OTU_334 (unclassified)	0.00000000	0.00073260	0.00000000	0.00043856	0.00017423	0.00047809
OTU_335 (unclassified)	0.00000000	0.00073260	0.00020796	0.00014619	0.00008712	0.00000000

OTU_336 (unclassified)	0.00000000	0.00000000	0.00041592	0.00000000	0.00000000	0.00000000
OTU_337 (Flavobacterium)	0.00000000	0.00008140	0.00034660	0.00000000	0.00034846	0.00007968
OTU_338 (unclassified)	0.00034449	0.00000000	0.00000000	0.00000000	0.00000000	0.00000000
OTU_339 (unclassified)	0.00000000	0.00000000	0.00034660	0.00000000	0.00017423	0.00000000
OTU_340 (TM7_genus_incertae_sedis)	0.00041339	0.00000000	0.00000000	0.00000000	0.00000000	0.00000000
OTU_341 (unclassified)	0.00006890	0.00089540	0.00041592	0.00073094	0.00000000	0.00031873
OTU_342 (unclassified)	0.00000000	0.00097680	0.00000000	0.00029238	0.00026135	0.00023904
OTU_343 (unclassified)	0.00000000	0.00000000	0.00090115	0.00014619	0.00000000	0.00007968
OTU_344 (unclassified)	0.00000000	0.00040700	0.00020796	0.00073094	0.00026135	0.00015936
OTU_345 (Granulicatella)	0.00000000	0.00000000	0.00076251	0.00000000	0.00017423	0.00000000
OTU_346 (unclassified)	0.00000000	0.00040700	0.00000000	0.00000000	0.00000000	0.00000000
OTU_347 (Aeromonas)	0.00027560	0.00000000	0.00041592	0.00219282	0.00531405	0.00191235
OTU_348 (unclassified)	0.00000000	0.00040700	0.00000000	0.00000000	0.00000000	0.00000000
OTU_349 (TM7_genus_incertae_sedis)	0.00000000	0.00000000	0.00076251	0.00000000	0.00000000	0.00007968
OTU_350 (Bdellovibrio)	0.00000000	0.00024420	0.00055455	0.00000000	0.00008712	0.00000000
OTU_351 (TM7_genus_incertae_sedis)	0.00048229	0.00000000	0.00000000	0.00000000	0.00000000	0.00000000
OTU_352 (unclassified)	0.00000000	0.00040700	0.00000000	0.00000000	0.00000000	0.00000000
OTU_353 (Knoellia)	0.00000000	0.00000000	0.00062387	0.00014619	0.00008712	0.00000000
OTU_354 (unclassified)	0.00000000	0.00065120	0.00000000	0.00065785	0.00008712	0.00015936
OTU_355 (Chlorophyta)	0.00000000	0.00008140	0.00062387	0.00000000	0.00000000	0.00000000
OTU_356 (unclassified)	0.00000000	0.00000000	0.00041592	0.00000000	0.00017423	0.00000000
OTU_357	0.00055119	0.00000000	0.00000000	0.00000000	0.00000000	0.00000000
OTU_358 (Emticicia)	0.00000000	0.00016280	0.00034660	0.00014619	0.00000000	0.00007968
OTU_359 (TM7_genus_incertae_sedis)	0.00041339	0.00000000	0.00000000	0.00007309	0.00000000	0.00000000
OTU_360 (unclassified)	0.00034449	0.00000000	0.00000000	0.00000000	0.00000000	0.00000000
OTU_361 (unclassified)	0.00027560	0.00244200	0.00027728	0.00073094	0.00017423	0.00055777
OTU_362 (SR1_genus_incertae_sedis)	0.00006890	0.00122100	0.00034660	0.00080403	0.00017423	0.00007968
OTU_363 (unclassified)	0.00000000	0.00056980	0.00000000	0.00000000	0.00000000	0.00000000
OTU_364 (unclassified)	0.00000000	0.00040700	0.00062387	0.00014619	0.00000000	0.00000000
OTU_365 (Gp4)	0.00013780	0.00040700	0.00034660	0.00014619	0.00000000	0.00000000
OTU_366 (unclassified)	0.00000000	0.00040700	0.00000000	0.00051166	0.00008712	0.00007968
OTU_367 (unclassified)	0.00000000	0.00040700	0.00000000	0.00000000	0.00000000	0.00000000
OTU_368 (unclassified)	0.00048229	0.00000000	0.00000000	0.00000000	0.00000000	0.00000000
OTU_369 (Legionella)	0.00000000	0.00040700	0.00000000	0.00000000	0.00000000	0.00000000
OTU_370 (Sphingomonas)	0.00062009	0.00008140	0.00000000	0.00000000	0.00008712	0.00000000
OTU_371 (unclassified)	0.00000000	0.00089540	0.00069319	0.00000000	0.00000000	0.00015936
OTU_372 (unclassified)	0.00000000	0.00000000	0.00048523	0.00029238	0.00000000	0.00000000
OTU_373 (unclassified)	0.00013780	0.00000000	0.00013864	0.00007309	0.01306734	0.00079681
OTU_374 (unclassified)	0.00048229	0.00000000	0.00000000	0.00000000	0.00000000	0.00000000
OTU_375 (unclassified)	0.00006890	0.00138380	0.00000000	0.00000000	0.00000000	0.00000000
OTU_376 (Streptococcus)	0.00000000	0.00024420	0.00034660	0.00000000	0.00017423	0.00015936
OTU_377 (Pedobacter)	0.00000000	0.00056980	0.00000000	0.00014619	0.00000000	0.00000000
OTU_378 (Ilumatobacter)	0.00000000	0.00032560	0.00000000	0.00000000	0.00000000	0.00000000
OTU_379 (unclassified)	0.00000000	0.00032560	0.00000000	0.00000000	0.00000000	0.00000000
OTU_380 (unclassified)	0.00000000	0.00187220	0.00020796	0.00021928	0.00000000	0.00015936

OTU_381 (OD1_genus_incertae_sedis)	0.00000000	0.00056980	0.00000000	0.00000000	0.00000000	0.00000000
OTU_382 (unclassified)	0.00000000	0.00073260	0.00027728	0.00065785	0.00043558	0.00063745
OTU_383 (Leadbetterella)	0.00000000	0.00000000	0.00048523	0.00000000	0.00017423	0.00007968
OTU_384 (unclassified)	0.00000000	0.00081400	0.00000000	0.00000000	0.00000000	0.00000000
OTU_385 (Clostridium_sensu_stricto)	0.00000000	0.00000000	0.00000000	0.00877129	0.00113250	0.00000000
OTU_386 (unclassified)	0.00000000	0.00008140	0.00048523	0.00000000	0.00017423	0.00007968
OTU_387 (unclassified)	0.00000000	0.00032560	0.00000000	0.00000000	0.00000000	0.00000000
OTU_388 (Bacillariophyta)	0.00000000	0.00056980	0.00020796	0.00014619	0.00000000	0.00000000
OTU_389 (unclassified)	0.00000000	0.00000000	0.00000000	0.00000000	0.00008712	0.00039841
OTU_390 (TM7_genus_incertae_sedis)	0.00000000	0.00089540	0.00000000	0.00000000	0.00000000	0.00000000
OTU_391 (Ilumatobacter)	0.00337605	0.00000000	0.00000000	0.00000000	0.00000000	0.00000000
OTU_392 (unclassified)	0.00096459	0.00048840	0.00048523	0.00000000	0.00008712	0.00000000
OTU_393 (unclassified)	0.00000000	0.00122100	0.00027728	0.00000000	0.00000000	0.00007968
OTU_394 (TM7_genus_incertae_sedis)	0.00000000	0.00065120	0.00076251	0.00021928	0.00000000	0.00023904
OTU_395 (unclassified)	0.00000000	0.00073260	0.00124775	0.00000000	0.00000000	0.00015936
OTU_396 (unclassified)	0.00000000	0.00016280	0.00069319	0.00029238	0.00034846	0.00000000
OTU_397 (unclassified)	0.00000000	0.00008140	0.00027728	0.00058475	0.00000000	0.00015936
OTU_398 (unclassified)	0.00000000	0.00073260	0.00000000	0.00000000	0.00017423	0.00000000
OTU_399 (unclassified)	0.00034449	0.00000000	0.00000000	0.00000000	0.00000000	0.00000000
OTU_400 (unclassified)	0.00000000	0.00040700	0.00041592	0.00073094	0.00017423	0.00023904
OTU_401 (unclassified)	0.00000000	0.00056980	0.00000000	0.00000000	0.00000000	0.00000000
OTU_402 (Rubrobacter)	0.00006890	0.00024420	0.00055455	0.00014619	0.00008712	0.00023904
OTU_403 (unclassified)	0.00068899	0.00000000	0.00000000	0.00000000	0.00000000	0.00000000
OTU_404 (Streptococcus)	0.00000000	0.00040700	0.00110911	0.00043856	0.00060981	0.00015936
OTU_405 (unclassified)	0.00000000	0.00000000	0.00034660	0.00029238	0.00017423	0.00063745
OTU_406 (unclassified)	0.00124018	0.00000000	0.00000000	0.00000000	0.00000000	0.00000000
OTU_407 (TM7_genus_incertae_sedis)	0.00048229	0.00040700	0.00034660	0.00021928	0.00000000	0.00007968
OTU_408 (unclassified)	0.00110238	0.00000000	0.00000000	0.00000000	0.00026135	0.00000000
OTU_409 (Aquicella)	0.00000000	0.00016280	0.00062387	0.00000000	0.00000000	0.00000000
OTU_410 (unclassified)	0.00000000	0.00000000	0.00027728	0.00000000	0.00000000	0.00000000
OTU_411 (TM7_genus_incertae_sedis)	0.00000000	0.00040700	0.00110911	0.00007309	0.00008712	0.00023904
OTU_412 (unclassified)	0.00062009	0.00000000	0.00000000	0.00000000	0.00000000	0.00000000
OTU_413 (unclassified)	0.00000000	0.00000000	0.00110911	0.00299686	0.00121962	0.00509960
OTU_414 (unclassified)	0.00000000	0.00081400	0.00000000	0.00000000	0.00000000	0.00000000
OTU_415 (unclassified)	0.00000000	0.00008140	0.00020796	0.00007309	0.00000000	0.00000000
OTU_416 (Armatimonas_Armatimonadetes_gp1)	0.00000000	0.00040700	0.00013864	0.00000000	0.00000000	0.00047809
OTU_417 (unclassified)	0.00000000	0.00016280	0.00041592	0.00000000	0.00000000	0.00007968
OTU_418 (Clostridium_sensu_stricto)	0.00000000	0.00138380	0.00000000	0.00000000	0.00008712	0.00000000
OTU_419 (unclassified)	0.00006890	0.00008140	0.00041592	0.00007309	0.00000000	0.00000000
OTU_420 (unclassified)	0.00000000	0.00056980	0.00000000	0.00000000	0.00000000	0.00000000
OTU_421 (Fluviicola)	0.00000000	0.00130240	0.00083183	0.00007309	0.00060981	0.00015936
OTU_422 (unclassified)	0.00000000	0.00000000	0.00027728	0.00021928	0.00000000	0.00000000

OTU_423 (unclassified)	0.00000000	0.00056980	0.00027728	0.00021928	0.00000000	0.00007968
OTU_424 (unclassified)	0.00000000	0.00056980	0.00000000	0.00000000	0.00000000	0.00000000
OTU_425 (unclassified)	0.00000000	0.00048840	0.00000000	0.00000000	0.00017423	0.00015936
OTU_426 (Janibacter)	0.00000000	0.00056980	0.00076251	0.00073094	0.00008712	0.00119522
OTU_427 (unclassified)	0.00068899	0.00000000	0.00000000	0.00036547	0.00000000	0.00000000
OTU_428 (unclassified)	0.00000000	0.00073260	0.00006932	0.00000000	0.00008712	0.00023904
OTU_429 (Kingella)	0.00013780	0.00000000	0.00062387	0.00014619	0.00017423	0.00023904
OTU_430 (Aquicella)	0.00034449	0.00000000	0.00000000	0.00000000	0.00000000	0.00000000
OTU_431 (Aeromonas)	0.00006890	0.00000000	0.00291141	0.00438564	0.00531405	0.00183267
OTU_432 (unclassified)	0.00000000	0.00056980	0.00013864	0.00058475	0.00008712	0.00047809
OTU_433 (unclassified)	0.00000000	0.00154660	0.00000000	0.00029238	0.00017423	0.00023904
OTU_434 (unclassified)	0.00000000	0.00000000	0.00048523	0.00000000	0.00000000	0.00031873
OTU_435 (unclassified)	0.00000000	0.00000000	0.00027728	0.00029238	0.00000000	0.00007968
OTU_436 (Mucilaginibacter)	0.00000000	0.00032560	0.00034660	0.00036547	0.00000000	0.00000000
OTU_437 (unclassified)	0.00000000	0.00000000	0.00034660	0.00000000	0.00000000	0.00000000
OTU_438 (unclassified)	0.00000000	0.00081400	0.00041592	0.00000000	0.00000000	0.00007968
OTU_439 (unclassified)	0.00000000	0.00000000	0.00020796	0.00000000	0.00000000	0.00000000
OTU_440 (unclassified)	0.00000000	0.00081400	0.00027728	0.00029238	0.00000000	0.00000000
OTU_441 (unclassified)	0.00000000	0.00000000	0.00041592	0.00000000	0.00000000	0.00000000
OTU_442 (TM7_genus_incertae_sedis)	0.00000000	0.00105820	0.00000000	0.00007309	0.00008712	0.00000000
OTU_443 (unclassified)	0.00000000	0.00089540	0.00000000	0.00014619	0.00000000	0.00015936
OTU_444 (unclassified)	0.00000000	0.00032560	0.00000000	0.00000000	0.00000000	0.00000000
OTU_445 (Flavobacterium)	0.00000000	0.00073260	0.00000000	0.00000000	0.00000000	0.00000000
OTU_446 (unclassified)	0.00000000	0.00024420	0.00000000	0.00000000	0.00000000	0.00000000
OTU_447 (unclassified)	0.00000000	0.00073260	0.00000000	0.00051166	0.00000000	0.00000000
OTU_448 (unclassified)	0.00000000	0.00032560	0.00000000	0.00000000	0.00000000	0.00000000
OTU_449 (unclassified)	0.00089569	0.00000000	0.00000000	0.00000000	0.00000000	0.00000000
OTU_450 (unclassified)	0.00000000	0.00040700	0.00000000	0.00000000	0.00000000	0.00000000
OTU_451 (unclassified)	0.00089569	0.00000000	0.00000000	0.00000000	0.00000000	0.00000000
OTU_452 (Methylotenera)	0.00000000	0.00016280	0.00034660	0.00000000	0.00000000	0.00007968
OTU_453 (unclassified)	0.00000000	0.00024420	0.00006932	0.00014619	0.00017423	0.00000000
OTU_454 (unclassified)	0.00000000	0.00048840	0.00000000	0.00000000	0.00000000	0.00000000
OTU_455 (Legionella)	0.00000000	0.00048840	0.00069319	0.00000000	0.00000000	0.00000000
OTU_456 (unclassified)	0.00096459	0.00000000	0.00000000	0.00000000	0.00000000	0.00000000
OTU_457 (unclassified)	0.00000000	0.00048840	0.00034660	0.00043856	0.00008712	0.00000000
OTU_458 (unclassified)	0.00000000	0.00024420	0.00000000	0.00000000	0.00000000	0.00000000
OTU_459 (unclassified)	0.00000000	0.00056980	0.00000000	0.00021928	0.00008712	0.00000000
OTU_460 (unclassified)	0.00000000	0.00040700	0.00020796	0.00000000	0.00000000	0.00000000
OTU_461 (unclassified)	0.00000000	0.00008140	0.00000000	0.00036547	0.00000000	0.00000000
OTU_462 (unclassified)	0.00000000	0.00040700	0.00000000	0.00000000	0.00000000	0.00000000
OTU_463 (unclassified)	0.00000000	0.00138380	0.00000000	0.00000000	0.00008712	0.00000000
OTU_464 (unclassified)	0.00000000	0.00000000	0.00020796	0.00000000	0.00000000	0.00000000
OTU_465 (unclassified)	0.00027560	0.00000000	0.00000000	0.00000000	0.00000000	0.00000000
OTU_466 (Flavobacterium)	0.00000000	0.00024420	0.00034660	0.00000000	0.00008712	0.00007968
OTU_467 (Methylobacterium)	0.00006890	0.00032560	0.00000000	0.00000000	0.00000000	0.00000000
OTU_468 (Brachybacterium)	0.00000000	0.00000000	0.00041592	0.00065785	0.00000000	0.00007968
OTU_469 (Gordonia)	0.00000000	0.00000000	0.00055455	0.00029238	0.00008712	0.00023904
OTU_470 (unclassified)	0.00034449	0.00000000	0.00000000	0.00000000	0.00000000	0.00000000
OTU_471 (unclassified)	0.00000000	0.00073260	0.00000000	0.00007309	0.00069692	0.00007968
OTU_472 (Flavobacterium)	0.00000000	0.00000000	0.00027728	0.00000000	0.00000000	0.00000000

OTU_473 (unclassified)	0.00082679	0.00000000	0.00000000	0.00000000	0.00000000	0.00000000
OTU_474 (unclassified)	0.00000000	0.00056980	0.00000000	0.00000000	0.00000000	0.00000000
OTU_475 (Sphingopyxis)	0.00000000	0.00032560	0.00076251	0.00000000	0.00008712	0.00055777
OTU_476 (unclassified)	0.00041339	0.00000000	0.00000000	0.00000000	0.00000000	0.00000000
OTU_477 (Micrococcus)	0.00020670	0.00032560	0.00000000	0.00000000	0.00000000	0.00000000
OTU_478 (Streptomyces)	0.00000000	0.00000000	0.00034660	0.00014619	0.00095827	0.00023904
OTU_479 (unclassified)	0.00020670	0.00000000	0.00000000	0.00000000	0.00000000	0.00000000
OTU_480 (Legionella)	0.00041339	0.00000000	0.00000000	0.00000000	0.00000000	0.00000000
OTU_481 (unclassified)	0.00000000	0.00040700	0.00000000	0.00000000	0.00000000	0.00000000
OTU_482 (Schlegelella)	0.00000000	0.00040700	0.00000000	0.00000000	0.00000000	0.00015936
OTU_483 (unclassified)	0.00000000	0.00000000	0.00041592	0.00000000	0.00000000	0.00039841
OTU_484 (Tetrasphaera)	0.00000000	0.00000000	0.00034660	0.00014619	0.00000000	0.00000000
OTU_485 (unclassified)	0.00372055	0.00000000	0.00000000	0.00021928	0.00008712	0.00015936
OTU_486 (unclassified)	0.00000000	0.00032560	0.00000000	0.00014619	0.00000000	0.00000000
OTU_487 (Flavobacterium)	0.00000000	0.00008140	0.00048523	0.00007309	0.00008712	0.00031873
OTU_488 (OD1_genus_incertae_sedis)	0.00006890	0.00195360	0.00110911	0.00000000	0.00026135	0.00007968
OTU_489 (unclassified)	0.00000000	0.00040700	0.00000000	0.00029238	0.00008712	0.00007968
OTU_490 (unclassified)	0.00027560	0.00000000	0.00000000	0.00000000	0.00000000	0.00000000
OTU_491 (OD1_genus_incertae_sedis)	0.00000000	0.00024420	0.00000000	0.00000000	0.00000000	0.00000000
OTU_492 (unclassified)	0.00068899	0.00016280	0.00000000	0.00000000	0.00000000	0.00000000
OTU_493 (Legionella)	0.00041339	0.00000000	0.00000000	0.00000000	0.00000000	0.00000000
OTU_494 (Paenibacillus)	0.00000000	0.00000000	0.00000000	0.00124260	0.00174231	0.00326693
OTU_495 (unclassified)	0.00000000	0.00000000	0.00055455	0.00000000	0.00000000	0.00007968
OTU_496 (unclassified)	0.00000000	0.00000000	0.00020796	0.00000000	0.00000000	0.00000000
OTU_497 (unclassified)	0.00000000	0.00154660	0.00041592	0.00219282	0.00156808	0.00087649
OTU_498 (unclassified)	0.00027560	0.00000000	0.00000000	0.00000000	0.00000000	0.00000000
OTU_499 (unclassified)	0.00000000	0.00008140	0.00027728	0.00021928	0.00008712	0.00023904
OTU_500 (Geobacillus)	0.00000000	0.00000000	0.00000000	0.00233901	0.00139385	0.00302789
OTU_501 (Corynebacterium)	0.00000000	0.00000000	0.00020796	0.00000000	0.00000000	0.00007968
OTU_502 (unclassified)	0.00000000	0.00056980	0.00000000	0.00000000	0.00000000	0.00000000
OTU_503 (unclassified)	0.00000000	0.00040700	0.00034660	0.00138879	0.00078404	0.00015936
OTU_504 (Nocardioides)	0.00096459	0.00048840	0.00048523	0.00000000	0.00008712	0.00031873
OTU_505 (TM7_genus_incertae_sedis)	0.00041339	0.00008140	0.00000000	0.00000000	0.00000000	0.00007968
OTU_506 (Rhodococcus)	0.00020670	0.00000000	0.00000000	0.00000000	0.00000000	0.00000000
OTU_507 (unclassified)	0.00020670	0.00000000	0.00000000	0.00000000	0.00000000	0.00000000
OTU_508 (Patulibacter)	0.00000000	0.00000000	0.00027728	0.00000000	0.00008712	0.00047809
OTU_509 (unclassified)	0.00000000	0.00000000	0.00055455	0.00000000	0.00008712	0.00063745
OTU_510 (Chlorophyta)	0.00000000	0.00040700	0.00090115	0.00000000	0.00008712	0.00023904
OTU_511 (unclassified)	0.00000000	0.00040700	0.00000000	0.00000000	0.00000000	0.00000000
OTU_512 (unclassified)	0.00000000	0.00000000	0.00013864	0.00000000	0.00008712	0.00007968
OTU_513 (TM7_genus_incertae_sedis)	0.00013780	0.00056980	0.00000000	0.00000000	0.00000000	0.00000000
OTU_514 (GpI)	0.00000000	0.00073260	0.00006932	0.00051166	0.00026135	0.00000000
OTU_515 (unclassified)	0.00000000	0.00073260	0.00069319	0.00080403	0.00008712	0.00039841
OTU_516 (Phenylobacterium)	0.00000000	0.00000000	0.00000000	0.00036547	0.00017423	0.00000000
OTU_517 (unclassified)	0.00020670	0.00154660	0.00090115	0.00021928	0.00000000	0.00031873
OTU_518 (Gemmatimonas)	0.00000000	0.00032560	0.00020796	0.00000000	0.00000000	0.00007968
OTU_519 (unclassified)	0.00034449	0.00000000	0.00000000	0.00000000	0.00000000	0.00000000

OTU_520 (Nesterenkonia)	0.00000000	0.00000000	0.00048523	0.00014619	0.00000000	0.00015936
OTU_521 (Brevundimonas)	0.00062009	0.00000000	0.00000000	0.00014619	0.00008712	0.00000000
OTU_522 (Gp4)	0.00000000	0.00056980	0.00013864	0.00014619	0.00017423	0.00000000
OTU_523 (unclassified)	0.00075789	0.00000000	0.00000000	0.00007309	0.00000000	0.00000000
OTU_524 (unclassified)	0.00000000	0.00024420	0.00000000	0.00000000	0.00000000	0.00000000
OTU_525 (unclassified)	0.00000000	0.00032560	0.00034660	0.00065785	0.00000000	0.00039841
OTU_526 (unclassified)	0.00000000	0.00073260	0.00000000	0.00000000	0.00000000	0.00000000
OTU_527 (unclassified)	0.00000000	0.00000000	0.00000000	0.00000000	0.00008712	0.00023904
OTU_528 (unclassified)	0.00020670	0.00032560	0.00027728	0.00073094	0.00043558	0.00055777
OTU_529 (unclassified)	0.00000000	0.00000000	0.00020796	0.00000000	0.00000000	0.00000000
OTU_530 (Carnobacterium)	0.00055119	0.00000000	0.00000000	0.00007309	0.00000000	0.00000000
OTU_531 (Variovorax)	0.00000000	0.00244200	0.00069319	0.00051166	0.00078404	0.00071713
OTU_532 (Aquicella)	0.00000000	0.00024420	0.00000000	0.00000000	0.00000000	0.00000000
OTU_533 (Chlorophyta)	0.00000000	0.00056980	0.00020796	0.00000000	0.00000000	0.00007968
OTU_534 (unclassified)	0.00034449	0.00000000	0.00000000	0.00000000	0.00000000	0.00000000
OTU_535 (Gp16)	0.00027560	0.00000000	0.00000000	0.00014619	0.00000000	0.00000000
OTU_536 (unclassified)	0.00055119	0.00000000	0.00006932	0.00000000	0.00000000	0.00000000
OTU_537 (TM7_genus_incertae_sedis)	0.00027560	0.00000000	0.00000000	0.00000000	0.00000000	0.00000000
OTU_538 (Streptophyta)	0.00682100	0.00032560	0.00000000	0.00058475	0.00026135	0.00031873
OTU_539 (unclassified)	0.00000000	0.00089540	0.00000000	0.00000000	0.00000000	0.00000000
OTU_540 (unclassified)	0.00048229	0.00000000	0.00000000	0.00000000	0.00000000	0.00000000
OTU_541 (unclassified)	0.00034449	0.00000000	0.00000000	0.00000000	0.00000000	0.00000000
OTU_542 (Brachybacterium)	0.00013780	0.00000000	0.00110911	0.00000000	0.00000000	0.00047809
OTU_543 (unclassified)	0.00000000	0.00024420	0.00000000	0.00000000	0.00000000	0.00000000
OTU_544 (unclassified)	0.00220477	0.00000000	0.00000000	0.00000000	0.00000000	0.00000000
OTU_545 (unclassified)	0.00000000	0.00056980	0.00041592	0.00014619	0.00026135	0.00047809
OTU_546 (unclassified)	0.00062009	0.00000000	0.00000000	0.00000000	0.00000000	0.00000000
OTU_547 (unclassified)	0.00000000	0.00016280	0.00000000	0.00014619	0.00034846	0.00015936
OTU_548 (Calditerricola)	0.00020670	0.00089540	0.00062387	0.00000000	0.00026135	0.00063745
OTU_549 (Ralstonia)	0.00000000	0.00000000	0.00055455	0.00043856	0.00000000	0.00000000
OTU_550 (unclassified)	0.00000000	0.00000000	0.00000000	0.00051166	0.00078404	0.00071713
OTU_551 (Corynebacterium)	0.00000000	0.00040700	0.00076251	0.00065785	0.00060981	0.00111554
OTU_552 (Paracoccus)	0.00013780	0.00016280	0.00000000	0.00000000	0.00000000	0.00000000
OTU_553 (unclassified)	0.00075789	0.00000000	0.00000000	0.00000000	0.00008712	0.00000000
OTU_554 (Sphaerotilus)	0.00000000	0.00073260	0.00000000	0.00000000	0.00000000	0.00007968
OTU_555 (unclassified)	0.00000000	0.00032560	0.00006932	0.00000000	0.00026135	0.00000000
OTU_556 (unclassified)	0.00000000	0.00048840	0.00000000	0.00000000	0.00000000	0.00000000
OTU_557 (unclassified)	0.00000000	0.00024420	0.00000000	0.00000000	0.00000000	0.00000000
OTU_558 (unclassified)	0.00000000	0.00000000	0.00027728	0.00000000	0.00000000	0.00007968
OTU_559 (unclassified)	0.00000000	0.00000000	0.00041592	0.00014619	0.00034846	0.00031873
OTU_560 (unclassified)	0.00000000	0.00032560	0.00027728	0.00000000	0.00008712	0.00031873
OTU_561 (Haliscomenobacter)	0.00000000	0.00073260	0.00013864	0.00000000	0.00008712	0.00023904
OTU_562 (unclassified)	0.00075789	0.00000000	0.00000000	0.00000000	0.00000000	0.00000000
OTU_563 (unclassified)	0.00000000	0.00040700	0.00000000	0.00000000	0.00000000	0.00000000
OTU_564 (Geothrix)	0.00000000	0.00065120	0.00000000	0.00000000	0.00000000	0.00000000
OTU_565 (unclassified)	0.00034449	0.00000000	0.00000000	0.00000000	0.00000000	0.00000000
OTU_566 (unclassified)	0.00000000	0.00000000	0.00027728	0.00000000	0.00000000	0.00000000
OTU_567 (unclassified)	0.00020670	0.00000000	0.00000000	0.00000000	0.00000000	0.00000000
OTU_568 (Aquicella)	0.00020670	0.00000000	0.00000000	0.00000000	0.00000000	0.00000000
OTU_569 (Meiothermus)	0.00034449	0.00000000	0.00000000	0.00000000	0.00000000	0.00000000

OTU_570 (OD1_genus_incertae_sedis)	0.00000000	0.00089540	0.00000000	0.00000000	0.00000000	0.00000000
OTU_571 (Dermacoccus)	0.00000000	0.00048840	0.00000000	0.00007309	0.00000000	0.00000000
OTU_572 (unclassified)	0.00000000	0.00008140	0.00034660	0.00000000	0.00000000	0.00015936
OTU_573 (TM7_genus_incertae_sedis)	0.00048229	0.00000000	0.00000000	0.00007309	0.00000000	0.00000000
OTU_574 (unclassified)	0.00075789	0.00000000	0.00000000	0.00000000	0.00000000	0.00000000
OTU_575 (unclassified)	0.00000000	0.00048840	0.00027728	0.00007309	0.00026135	0.00063745
OTU_576 (unclassified)	0.00000000	0.00040700	0.00000000	0.00000000	0.00000000	0.00000000
OTU_577 (Iamia)	0.00000000	0.00024420	0.00000000	0.00007309	0.00000000	0.00000000
OTU_578 (Singulisphaera)	0.00000000	0.00000000	0.00000000	0.00080403	0.00000000	0.00000000
OTU_579 (OD1_genus_incertae_sedis)	0.00000000	0.00073260	0.00000000	0.00000000	0.00000000	0.00000000
OTU_580 (unclassified)	0.00000000	0.00048840	0.00041592	0.00000000	0.00000000	0.00000000
OTU_581 (TM7_genus_incertae_sedis)	0.00000000	0.00032560	0.00000000	0.00000000	0.00000000	0.00000000
OTU_582 (TM7_genus_incertae_sedis)	0.00000000	0.00008140	0.00048523	0.00014619	0.00000000	0.00000000
OTU_583 (unclassified)	0.00000000	0.00024420	0.00000000	0.00124260	0.00052269	0.00039841
OTU_584 (Haliscomenobacter)	0.00000000	0.00081400	0.00000000	0.00021928	0.00000000	0.00000000
OTU_585 (unclassified)	0.00000000	0.00056980	0.00020796	0.00000000	0.00026135	0.00071713
OTU_586 (unclassified)	0.00000000	0.00000000	0.00076251	0.00029238	0.00017423	0.00007968
OTU_587 (unclassified)	0.00000000	0.00032560	0.00000000	0.00000000	0.00000000	0.00000000
OTU_588 (unclassified)	0.00000000	0.00048840	0.00000000	0.00000000	0.00000000	0.00000000
OTU_589 (Bdellovibrio)	0.00000000	0.00024420	0.00020796	0.00000000	0.00000000	0.00000000
OTU_590 (unclassified)	0.00000000	0.00032560	0.00000000	0.00000000	0.00000000	0.00000000
OTU_591 (unclassified)	0.00000000	0.00040700	0.00020796	0.00000000	0.00000000	0.00007968
OTU_592 (unclassified)	0.00000000	0.00032560	0.00000000	0.00000000	0.00000000	0.00000000
OTU_593 (unclassified)	0.00034449	0.00000000	0.00000000	0.00000000	0.00000000	0.00000000
OTU_594 (unclassified)	0.00000000	0.00024420	0.00000000	0.00007309	0.00000000	0.00000000
OTU_595 (Capnocytophaga)	0.00000000	0.00000000	0.00034660	0.00000000	0.00000000	0.00015936
OTU_596 (Iamia)	0.00048229	0.00024420	0.00000000	0.00000000	0.00000000	0.00000000
OTU_597 (TM7_genus_incertae_sedis)	0.00000000	0.00024420	0.00000000	0.00000000	0.00000000	0.00000000
OTU_598 (unclassified)	0.00000000	0.00048840	0.00000000	0.00000000	0.00000000	0.00000000
OTU_599 (unclassified)	0.00082679	0.00000000	0.00000000	0.00000000	0.00000000	0.00000000
OTU_600 (unclassified)	0.00000000	0.00040700	0.00000000	0.00000000	0.00000000	0.00000000
OTU_601 (Bacillariophyta)	0.00000000	0.00008140	0.00048523	0.00007309	0.00000000	0.00007968
OTU_602 (Gp4)	0.00000000	0.00024420	0.00000000	0.00000000	0.00000000	0.00000000
OTU_603 (unclassified)	0.00000000	0.00000000	0.00020796	0.00007309	0.00000000	0.00000000
OTU_604 (unclassified)	0.00000000	0.00000000	0.00034660	0.00007309	0.00000000	0.00015936
OTU_605 (unclassified)	0.00000000	0.00040700	0.00000000	0.00000000	0.00000000	0.00000000
OTU_606 (TM7_genus_incertae_sedis)	0.00000000	0.00105820	0.00000000	0.00000000	0.00000000	0.00000000
OTU_607 (unclassified)	0.00000000	0.00065120	0.00000000	0.00000000	0.00000000	0.00000000
OTU_608 (unclassified)	0.00000000	0.00000000	0.00034660	0.00000000	0.00000000	0.00000000
OTU_609 (unclassified)	0.00000000	0.00040700	0.00006932	0.00000000	0.00000000	0.00000000
OTU_610 (unclassified)	0.00000000	0.00073260	0.00000000	0.00000000	0.00000000	0.00007968
OTU_611 (unclassified)	0.00000000	0.00040700	0.00062387	0.00007309	0.00008712	0.00023904
OTU_612 (Paludibacter)	0.00000000	0.00040700	0.00000000	0.00000000	0.00000000	0.00000000
OTU_613 (unclassified)	0.00000000	0.00000000	0.00000000	0.00021928	0.00017423	0.00031873

OTU_614 (unclassified)	0.00000000	0.00065120	0.00000000	0.00000000	0.00000000	0.00000000
OTU_615 (unclassified)	0.00000000	0.00032560	0.00034660	0.00043856	0.00034846	0.00023904
OTU_616 (OD1_genus_incertae_sedis)	0.00000000	0.00032560	0.00006932	0.00000000	0.00000000	0.00007968
OTU_617 (unclassified)	0.00000000	0.00032560	0.00000000	0.00021928	0.00008712	0.00015936
OTU_618 (unclassified)	0.00062009	0.00000000	0.00000000	0.00000000	0.00000000	0.00000000
OTU_619 (Paracoccus)	0.00013780	0.00000000	0.00090115	0.00036547	0.00043558	0.00047809
OTU_620 (Bacillariophyta)	0.00000000	0.00032560	0.00055455	0.00000000	0.00000000	0.00015936
OTU_621 (unclassified)	0.00000000	0.00000000	0.00000000	0.00021928	0.00348462	0.00000000
OTU_622 (Carboxydocella)	0.00048229	0.00000000	0.00006932	0.00000000	0.00034846	0.00000000
OTU_623 (OD1_genus_incertae_sedis)	0.00000000	0.00024420	0.00013864	0.00029238	0.00000000	0.00000000
OTU_624 (OD1_genus_incertae_sedis)	0.00000000	0.00211640	0.00048523	0.00131569	0.00008712	0.00015936
OTU_625 (unclassified)	0.00000000	0.00024420	0.00000000	0.00000000	0.00000000	0.00000000
OTU_626 (OD1_genus_incertae_sedis)	0.00000000	0.00000000	0.00041592	0.00000000	0.00000000	0.00015936
OTU_627 (Gp3)	0.00000000	0.00016280	0.00027728	0.00000000	0.00000000	0.00000000
OTU_628 (unclassified)	0.00048229	0.00000000	0.00000000	0.00000000	0.00000000	0.00000000
OTU_629 (Propionibacterium)	0.00020670	0.00024420	0.00000000	0.00000000	0.00000000	0.00000000
OTU_630 (Kocuria)	0.00000000	0.00000000	0.00069319	0.00000000	0.00000000	0.00000000
OTU_631 (unclassified)	0.00013780	0.00000000	0.00000000	0.00000000	0.00000000	0.00000000
OTU_632 (Tetragenococcus)	0.00020670	0.00113960	0.00131707	0.00299686	0.00200366	0.00191235
OTU_633 (unclassified)	0.00041339	0.00000000	0.00000000	0.00000000	0.00000000	0.00000000
OTU_634 (Pedomicrobium)	0.00130908	0.00016280	0.00000000	0.00000000	0.00000000	0.00000000
OTU_635 (unclassified)	0.00020670	0.00000000	0.00000000	0.00000000	0.00000000	0.00000000
OTU_636 (unclassified)	0.00027560	0.00000000	0.00000000	0.00000000	0.00000000	0.00000000
OTU_637 (unclassified)	0.00034449	0.00000000	0.00000000	0.00000000	0.00000000	0.00000000
OTU_638 (unclassified)	0.00034449	0.00048840	0.00027728	0.00080403	0.00008712	0.00047809
OTU_639 (unclassified)	0.00055119	0.00000000	0.00000000	0.00051166	0.00000000	0.00000000
OTU_640 (Bosea)	0.00089569	0.00073260	0.00027728	0.00051166	0.00087116	0.00079681
OTU_641 (unclassified)	0.00013780	0.00024420	0.00000000	0.00000000	0.00000000	0.00000000
OTU_642 (unclassified)	0.00000000	0.00000000	0.00000000	0.00241210	0.00000000	0.00000000
OTU_643 (Paenibacillus)	0.00000000	0.00000000	0.00000000	0.00197354	0.00078404	0.00191235
OTU_644 (unclassified)	0.00000000	0.00000000	0.00000000	0.00007309	0.00008712	0.00000000
OTU_645 (unclassified)	0.00020670	0.00000000	0.00000000	0.00007309	0.00000000	0.00000000
OTU_646 (Ochrobactrum)	0.00006890	0.00065120	0.00110911	0.00131569	0.00217789	0.00191235
OTU_647 (unclassified)	0.00055119	0.00000000	0.00000000	0.00000000	0.00000000	0.00000000
OTU_648 (Halomonas)	0.00006890	0.00008140	0.00013864	0.00043856	0.00026135	0.00007968
OTU_649 (unclassified)	0.00034449	0.00016280	0.00000000	0.00000000	0.00000000	0.00000000
OTU_650 (unclassified)	0.00020670	0.00000000	0.00000000	0.00000000	0.00000000	0.00000000
OTU_651 (unclassified)	0.00137798	0.00000000	0.00000000	0.00000000	0.00000000	0.00000000
OTU_652 (unclassified)	0.00034449	0.00000000	0.00000000	0.00000000	0.00000000	0.00000000
OTU_653 (unclassified)	0.00000000	0.00000000	0.00000000	0.00000000	0.00043558	0.00039841
OTU_654 (Hyphomicrobium)	0.00000000	0.00024420	0.00006932	0.00036547	0.00017423	0.00047809
OTU_655 (unclassified)	0.00034449	0.00000000	0.00000000	0.00000000	0.00000000	0.00000000
OTU_656 (Legionella)	0.00013780	0.00000000	0.00000000	0.00000000	0.00000000	0.00000000
OTU_657 (Iamia)	0.00062009	0.00000000	0.00000000	0.00000000	0.00000000	0.00000000
OTU_658 (Leucobacter)	0.00013780	0.00008140	0.00000000	0.00000000	0.00000000	0.00000000
OTU_659 (Ilumatobacter)	0.00000000	0.00000000	0.00013864	0.00029238	0.00026135	0.00071713
OTU_660 (unclassified)	0.00027560	0.00000000	0.00000000	0.00000000	0.00000000	0.00000000

OTU_661 (Gp5)	0.00020670	0.00000000	0.00000000	0.00000000	0.00000000	0.00000000
OTU_662 (unclassified)	0.00013780	0.00000000	0.00000000	0.00000000	0.00000000	0.00000000
OTU_663 (unclassified)	0.00020670	0.00000000	0.00006932	0.00000000	0.00000000	0.00023904
OTU_664 (unclassified)	0.00020670	0.00000000	0.00000000	0.00000000	0.00000000	0.00000000
OTU_665 (Delftia)	0.00000000	0.00008140	0.00041592	0.00204663	0.00017423	0.00079681
OTU_666 (unclassified)	0.00000000	0.00000000	0.00034660	0.00021928	0.00000000	0.00000000
OTU_667 (Aquicella)	0.00020670	0.00000000	0.00000000	0.00000000	0.00000000	0.00000000
OTU_668 (unclassified)	0.00013780	0.00000000	0.00000000	0.00000000	0.00000000	0.00000000
OTU_669 (unclassified)	0.00013780	0.00000000	0.00000000	0.00000000	0.00000000	0.00000000
OTU_670 (Thiothrix)	0.00000000	0.00000000	0.00000000	0.00095022	0.00008712	0.00000000
OTU_671 (unclassified)	0.00068899	0.00000000	0.00000000	0.00000000	0.00000000	0.00000000
OTU_672 (unclassified)	0.00000000	0.00000000	0.00000000	0.00029238	0.00026135	0.00031873
OTU_673 (unclassified)	0.00027560	0.00032560	0.00000000	0.00000000	0.00000000	0.00000000
OTU_674 (unclassified)	0.00000000	0.00000000	0.00000000	0.00153498	0.00043558	0.00000000
OTU_675 (Aquicella)	0.00020670	0.00000000	0.00000000	0.00000000	0.00000000	0.00000000
OTU_676 (Aquincola)	0.00000000	0.00056980	0.00055455	0.00095022	0.00069692	0.00055777
OTU_677 (TM7_genus_incertae_sedis)	0.00000000	0.00016280	0.00013864	0.00029238	0.00000000	0.00000000
OTU_678 (unclassified)	0.00041339	0.00000000	0.00000000	0.00000000	0.00000000	0.00000000
OTU_679 (Nocardioides)	0.00020670	0.00000000	0.00000000	0.00000000	0.00000000	0.00000000
OTU_680 (Bdellovibrio)	0.00000000	0.00008140	0.00027728	0.00007309	0.00000000	0.00007968
OTU_681 (unclassified)	0.00013780	0.00000000	0.00027728	0.00000000	0.00008712	0.00000000
OTU_682 (unclassified)	0.00013780	0.00000000	0.00000000	0.00000000	0.00000000	0.00000000
OTU_683 (Lactobacillus)	0.00000000	0.00000000	0.00000000	0.00431255	0.00200366	0.00000000
OTU_684 (unclassified)	0.00000000	0.00056980	0.00020796	0.00000000	0.00008712	0.00000000
OTU_685 (unclassified)	0.00000000	0.00016280	0.00000000	0.00014619	0.00000000	0.00000000
OTU_686 (unclassified)	0.00000000	0.00056980	0.00000000	0.00014619	0.00008712	0.00023904
OTU_687 (unclassified)	0.00000000	0.00016280	0.00000000	0.00000000	0.00000000	0.00000000
OTU_688 (unclassified)	0.00000000	0.00048840	0.00000000	0.00000000	0.00000000	0.00000000
OTU_689 (Methyloversatilis)	0.00000000	0.00016280	0.00041592	0.00000000	0.00034846	0.00007968
OTU_690 (unclassified)	0.00000000	0.00040700	0.00000000	0.00000000	0.00000000	0.00000000
OTU_691 (unclassified)	0.00000000	0.00000000	0.00020796	0.00000000	0.00000000	0.00000000
OTU_692 (unclassified)	0.00000000	0.00000000	0.00027728	0.00000000	0.00000000	0.00000000
OTU_693 (unclassified)	0.00000000	0.00032560	0.00000000	0.00000000	0.00000000	0.00000000
OTU_694 (unclassified)	0.00000000	0.00008140	0.00006932	0.00000000	0.00008712	0.00000000
OTU_695 (unclassified)	0.00000000	0.00000000	0.00013864	0.00000000	0.00000000	0.00000000
OTU_696 (unclassified)	0.00000000	0.00016280	0.00000000	0.00000000	0.00000000	0.00000000
OTU_697 (unclassified)	0.00000000	0.00000000	0.00027728	0.00000000	0.00000000	0.00000000
OTU_698 (unclassified)	0.00000000	0.00000000	0.00000000	0.00000000	0.00017423	0.00000000
OTU_699 (unclassified)	0.00000000	0.00016280	0.00000000	0.00000000	0.00000000	0.00000000
OTU_700 (unclassified)	0.00000000	0.00000000	0.00020796	0.00000000	0.00000000	0.00000000
OTU_701 (Vogesella)	0.00000000	0.00000000	0.00034660	0.00007309	0.00008712	0.00023904
OTU_702 (unclassified)	0.00000000	0.00089540	0.00013864	0.00000000	0.00000000	0.00000000
OTU_703 (Bacillariophyta)	0.00006890	0.00040700	0.00000000	0.00000000	0.00000000	0.00000000
OTU_704 (unclassified)	0.00000000	0.00008140	0.00027728	0.00000000	0.00000000	0.00007968
OTU_705 (unclassified)	0.00000000	0.00138380	0.00006932	0.00000000	0.00000000	0.00000000
OTU_706 (unclassified)	0.00020670	0.00000000	0.00000000	0.00000000	0.00000000	0.00000000
OTU_707 (Flavobacterium)	0.00027560	0.00000000	0.00000000	0.00000000	0.00000000	0.00000000
OTU_708 (unclassified)	0.00000000	0.00000000	0.00000000	0.00014619	0.00000000	0.00000000
OTU_709 (unclassified)	0.00013780	0.00000000	0.00000000	0.00000000	0.00000000	0.00000000
OTU_710 (Aquicella)	0.00020670	0.00000000	0.00000000	0.00000000	0.00000000	0.00000000

OTU_711 (Sandaracinobacter)	0.00062009	0.00000000	0.00000000	0.00000000	0.00000000	0.00000000
OTU_712 (Legionella)	0.00055119	0.00000000	0.00000000	0.00000000	0.00000000	0.00000000
OTU_713 (TM7_genus_incertae_sedis)	0.00041339	0.00000000	0.00000000	0.00000000	0.00000000	0.00000000
OTU_714 (unclassified)	0.00192917	0.00000000	0.00000000	0.00007309	0.00000000	0.00000000
OTU_715 (Patulibacter)	0.00027560	0.00000000	0.00000000	0.00029238	0.00000000	0.00000000
OTU_716 (unclassified)	0.00027560	0.00000000	0.00000000	0.00000000	0.00000000	0.00000000
OTU_717 (unclassified)	0.00000000	0.00000000	0.00013864	0.00000000	0.00000000	0.00023904
OTU_718 (Microbacterium)	0.00000000	0.00000000	0.00000000	0.00000000	0.00026135	0.00055777
OTU_719 (unclassified)	0.00117128	0.00000000	0.00000000	0.00000000	0.00000000	0.00000000
OTU_720 (Roseomonas)	0.00048229	0.00000000	0.00000000	0.00000000	0.00000000	0.00000000
OTU_721 (Legionella)	0.00020670	0.00000000	0.00000000	0.00000000	0.00000000	0.00000000
OTU_722 (Clostridium_XI)	0.00027560	0.00000000	0.00000000	0.00000000	0.00000000	0.00000000
OTU_723 (unclassified)	0.00013780	0.00000000	0.00000000	0.00000000	0.00000000	0.00000000
OTU_724 (Clostridium_sensu_stricto)	0.00516742	0.00000000	0.00000000	0.00000000	0.00000000	0.00000000
OTU_725 (unclassified)	0.00034449	0.00000000	0.00000000	0.00007309	0.00000000	0.00000000
OTU_726 (Clostridium_sensu_stricto)	0.00027560	0.00000000	0.00000000	0.00000000	0.00000000	0.00000000
OTU_727 (unclassified)	0.00020670	0.00000000	0.00000000	0.00000000	0.00000000	0.00000000
OTU_728 (Gp11)	0.00013780	0.00000000	0.00000000	0.00000000	0.00000000	0.00000000
OTU_729 (unclassified)	0.00013780	0.00040700	0.00027728	0.00000000	0.00008712	0.00000000
OTU_730 (unclassified)	0.00062009	0.00000000	0.00000000	0.00000000	0.00000000	0.00000000
OTU_731 (unclassified)	0.00000000	0.00048840	0.00000000	0.00000000	0.00008712	0.00000000
OTU_732 (unclassified)	0.00000000	0.00016280	0.00000000	0.00000000	0.00000000	0.00000000
OTU_733 (unclassified)	0.00000000	0.00162800	0.00097047	0.00051166	0.00069692	0.00031873
OTU_734 (unclassified)	0.00000000	0.00032560	0.00000000	0.00000000	0.00000000	0.00000000
OTU_735 (Corynebacterium)	0.00000000	0.00122100	0.00000000	0.00000000	0.00000000	0.00000000
OTU_736 (unclassified)	0.00000000	0.00040700	0.00020796	0.00014619	0.00000000	0.00000000
OTU_737 (unclassified)	0.00013780	0.00000000	0.00000000	0.00000000	0.00000000	0.00000000
OTU_738 (unclassified)	0.00027560	0.00000000	0.00000000	0.00000000	0.00000000	0.00000000
OTU_739 (Geobacillus)	0.00020670	0.00008140	0.00041592	0.00073094	0.00034846	0.00007968
OTU_740 (unclassified)	0.00034449	0.00000000	0.00000000	0.00000000	0.00000000	0.00000000
OTU_741 (unclassified)	0.00062009	0.00000000	0.00000000	0.00000000	0.00000000	0.00000000
OTU_742 (unclassified)	0.00020670	0.00000000	0.00000000	0.00000000	0.00000000	0.00000000
OTU_743 (unclassified)	0.00000000	0.00000000	0.00020796	0.00007309	0.00017423	0.00039841
OTU_744 (unclassified)	0.00027560	0.00000000	0.00000000	0.00000000	0.00000000	0.00000000
OTU_745 (unclassified)	0.00041339	0.00000000	0.00000000	0.00000000	0.00000000	0.00000000
OTU_746 (Legionella)	0.00034449	0.00000000	0.00000000	0.00014619	0.00000000	0.00007968
OTU_747 (Legionella)	0.00000000	0.00016280	0.00020796	0.00021928	0.00017423	0.00007968
OTU_748 (Aquicella)	0.00041339	0.00000000	0.00000000	0.00000000	0.00000000	0.00000000
OTU_749 (unclassified)	0.00048229	0.00000000	0.00000000	0.00000000	0.00000000	0.00000000
OTU_750 (unclassified)	0.00027560	0.00000000	0.00000000	0.00000000	0.00000000	0.00000000
OTU_751 (unclassified)	0.00096459	0.00000000	0.00000000	0.00000000	0.00000000	0.00000000
OTU_752 (unclassified)	0.00027560	0.00000000	0.00000000	0.00000000	0.00000000	0.00000000
OTU_753 (TM7_genus_incertae_sedis)	0.00027560	0.00000000	0.00000000	0.00000000	0.00000000	0.00000000
OTU_754 (unclassified)	0.00062009	0.00000000	0.00006932	0.00000000	0.00000000	0.00000000
OTU_755 (unclassified)	0.00013780	0.00000000	0.00000000	0.00000000	0.00000000	0.00000000
OTU_756 (unclassified)	0.00055119	0.00000000	0.00000000	0.00000000	0.00000000	0.00000000
OTU_757 (unclassified)	0.00062009	0.00000000	0.00000000	0.00000000	0.00000000	0.00000000

OTU_758 (unclassified)	0.00192917	0.00000000	0.00000000	0.00000000	0.00000000	0.00000000
OTU_759 (TM7_genus_incertae_sedis)	0.00000000	0.00000000	0.00000000	0.00087713	0.00000000	0.00007968
OTU_760 (unclassified)	0.00020670	0.00000000	0.00000000	0.00000000	0.00000000	0.00000000
OTU_761 (unclassified)	0.00013780	0.00000000	0.00000000	0.00000000	0.00000000	0.00000000
OTU_762 (unclassified)	0.00000000	0.00000000	0.00006932	0.00051166	0.00000000	0.00023904
OTU_763 (unclassified)	0.00013780	0.00000000	0.00000000	0.00000000	0.00000000	0.00000000
OTU_764 (unclassified)	0.00013780	0.00000000	0.00000000	0.00000000	0.00000000	0.00000000
OTU_765 (OD1_genus_incertae_sedis)	0.00000000	0.00000000	0.00000000	0.00043856	0.00000000	0.00007968
OTU_766 (unclassified)	0.00000000	0.00040700	0.00000000	0.00000000	0.00000000	0.00000000
OTU_767 (unclassified)	0.00000000	0.00000000	0.00000000	0.00007309	0.00008712	0.00007968
OTU_768 (Legionella)	0.00000000	0.00065120	0.00034660	0.00021928	0.00034846	0.00047809
OTU_769 (unclassified)	0.00000000	0.00089540	0.00000000	0.00000000	0.00000000	0.00000000
OTU_770 (unclassified)	0.00000000	0.00048840	0.00000000	0.00000000	0.00000000	0.00000000
OTU_771 (unclassified)	0.00000000	0.00016280	0.00000000	0.00000000	0.00000000	0.00000000
OTU_772 (unclassified)	0.00000000	0.00024420	0.00000000	0.00036547	0.00000000	0.00015936
OTU_773 (Weissella)	0.00000000	0.00000000	0.00000000	0.00065785	0.00365886	0.00000000
OTU_774 (TM7_genus_incertae_sedis)	0.00006890	0.00024420	0.00000000	0.00000000	0.00000000	0.00000000
OTU_775 (unclassified)	0.00000000	0.00048840	0.00006932	0.00000000	0.00000000	0.00000000
OTU_776 (unclassified)	0.00000000	0.00016280	0.00000000	0.00000000	0.00000000	0.00000000
OTU_777 (unclassified)	0.00000000	0.00024420	0.00000000	0.00000000	0.00000000	0.00000000
OTU_778 (unclassified)	0.00000000	0.00016280	0.00027728	0.00021928	0.00000000	0.00007968
OTU_779 (unclassified)	0.00000000	0.00032560	0.00000000	0.00000000	0.00000000	0.00000000
OTU_780 (unclassified)	0.00000000	0.00024420	0.00000000	0.00000000	0.00000000	0.00000000
OTU_781 (unclassified)	0.00000000	0.00048840	0.00013864	0.00000000	0.00008712	0.00000000
OTU_782 (unclassified)	0.00000000	0.00040700	0.00000000	0.00000000	0.00034846	0.00007968
OTU_783 (unclassified)	0.00000000	0.00016280	0.00000000	0.00000000	0.00000000	0.00000000
OTU_784 (unclassified)	0.00000000	0.00040700	0.00000000	0.00000000	0.00000000	0.00000000
OTU_785 (Legionella)	0.00000000	0.00000000	0.00006932	0.00014619	0.00000000	0.00031873
OTU_786 (unclassified)	0.00000000	0.00016280	0.00000000	0.00000000	0.00000000	0.00000000
OTU_787 (unclassified)	0.00000000	0.00056980	0.00069319	0.00219282	0.00200366	0.00127490
OTU_788 (unclassified)	0.00034449	0.00000000	0.00000000	0.00000000	0.00000000	0.00000000
OTU_789 (unclassified)	0.00013780	0.00097680	0.00000000	0.00029238	0.00034846	0.00007968
OTU_790 (Gp3)	0.00000000	0.00016280	0.00000000	0.00000000	0.00000000	0.00000000
OTU_791 (unclassified)	0.00006890	0.00032560	0.00000000	0.00007309	0.00000000	0.00000000
OTU_792 (unclassified)	0.00000000	0.00089540	0.00000000	0.00000000	0.00000000	0.00000000
OTU_793 (Bacteriovorax)	0.00000000	0.00024420	0.00006932	0.00000000	0.00000000	0.00000000
OTU_794 (Gluconobacter)	0.00000000	0.00000000	0.00000000	0.00007309	0.00060981	0.00000000
OTU_795 (unclassified)	0.00000000	0.00040700	0.00000000	0.00000000	0.00000000	0.00000000
OTU_796 (unclassified)	0.00000000	0.00016280	0.00000000	0.00000000	0.00000000	0.00000000
OTU_797 (TM7_genus_incertae_sedis)	0.00000000	0.00024420	0.00000000	0.00000000	0.00000000	0.00000000
OTU_798 (unclassified)	0.00000000	0.00000000	0.00013864	0.00051166	0.00000000	0.00000000
OTU_799 (unclassified)	0.00075789	0.00000000	0.00000000	0.00000000	0.00000000	0.00000000
OTU_800 (unclassified)	0.00020670	0.00024420	0.00000000	0.00000000	0.00000000	0.00000000
OTU_801 (Ilumatobacter)	0.00027560	0.00000000	0.00000000	0.00000000	0.00000000	0.00000000
OTU_802 (unclassified)	0.00027560	0.00000000	0.00000000	0.00000000	0.00000000	0.00000000
OTU_803 (unclassified)	0.00027560	0.00000000	0.00000000	0.00000000	0.00000000	0.00000000
OTU_804 (unclassified)	0.00048229	0.00000000	0.00000000	0.00000000	0.00000000	0.00000000

OTU_805 (unclassified)	0.00034449	0.00000000	0.00000000	0.00000000	0.00000000	0.00000000
OTU_806 (unclassified)	0.00000000	0.00000000	0.00000000	0.00021928	0.00008712	0.00007968
OTU_807 (unclassified)	0.00000000	0.00008140	0.00000000	0.00029238	0.00017423	0.00015936
OTU_808 (unclassified)	0.00027560	0.00000000	0.00000000	0.00000000	0.00000000	0.00000000
OTU_809 (unclassified)	0.00020670	0.00000000	0.00000000	0.00000000	0.00000000	0.00000000
OTU_810 (unclassified)	0.00055119	0.00000000	0.00000000	0.00000000	0.00000000	0.00000000
OTU_811 (unclassified)	0.00000000	0.00000000	0.00006932	0.00043856	0.00000000	0.00047809
OTU_812 (unclassified)	0.00013780	0.00000000	0.00000000	0.00000000	0.00000000	0.00000000
OTU_813 (unclassified)	0.00013780	0.00024420	0.00000000	0.00000000	0.00000000	0.00000000
OTU_814 (unclassified)	0.00034449	0.00000000	0.00000000	0.00000000	0.00000000	0.00000000
OTU_815 (Corynebacterium)	0.00000000	0.00016280	0.00020796	0.00058475	0.00008712	0.00055777
OTU_816 (unclassified)	0.00013780	0.00000000	0.00000000	0.00000000	0.00000000	0.00000000
OTU_817 (Aquicella)	0.00013780	0.00000000	0.00000000	0.00000000	0.00000000	0.00000000
OTU_818 (Clostridium_sensu_stricto)	0.00013780	0.00000000	0.00000000	0.00000000	0.00000000	0.00000000
OTU_819 (Legionella)	0.00013780	0.00000000	0.00000000	0.00000000	0.00000000	0.00000000
OTU_820 (unclassified)	0.00041339	0.00032560	0.00013864	0.00000000	0.00017423	0.00023904
OTU_821 (unclassified)	0.00041339	0.00000000	0.00000000	0.00000000	0.00000000	0.00000000
OTU_822 (Chryseobacterium)	0.00006890	0.00024420	0.00000000	0.00000000	0.00000000	0.00000000
OTU_823 (unclassified)	0.00103348	0.00000000	0.00000000	0.00000000	0.00000000	0.00000000
OTU_824 (unclassified)	0.00020670	0.00000000	0.00000000	0.00000000	0.00000000	0.00000000
OTU_825 (unclassified)	0.00013780	0.00000000	0.00000000	0.00000000	0.00000000	0.00000000
OTU_826 (unclassified)	0.00020670	0.00000000	0.00000000	0.00000000	0.00000000	0.00000000
OTU_827 (unclassified)	0.00013780	0.00000000	0.00000000	0.00000000	0.00000000	0.00000000
OTU_828 (unclassified)	0.00013780	0.00000000	0.00000000	0.00000000	0.00000000	0.00000000
OTU_829 (Roseomonas)	0.00013780	0.00000000	0.00000000	0.00000000	0.00000000	0.00000000
OTU_830 (Gp5)	0.00027560	0.00000000	0.00000000	0.00000000	0.00000000	0.00000000
OTU_831 (unclassified)	0.00034449	0.00000000	0.00000000	0.00021928	0.00000000	0.00000000
OTU_832 (unclassified)	0.00013780	0.00000000	0.00000000	0.00000000	0.00000000	0.00000000
OTU_833 (unclassified)	0.00013780	0.00000000	0.00000000	0.00000000	0.00000000	0.00000000
OTU_834 (unclassified)	0.00000000	0.00040700	0.00000000	0.00000000	0.00052269	0.00055777
OTU_835 (unclassified)	0.00034449	0.00000000	0.00000000	0.00000000	0.00000000	0.00000000
OTU_836 (Nocardioides)	0.00020670	0.00000000	0.00000000	0.00000000	0.00000000	0.00000000
OTU_837 (Legionella)	0.00013780	0.00000000	0.00000000	0.00000000	0.00000000	0.00000000
OTU_838 (Gp6)	0.00034449	0.00000000	0.00000000	0.00000000	0.00000000	0.00000000
OTU_839 (unclassified)	0.00344495	0.00000000	0.00000000	0.00051166	0.00017423	0.00000000
OTU_840 (unclassified)	0.00013780	0.00000000	0.00000000	0.00000000	0.00000000	0.00000000
OTU_841 (unclassified)	0.00027560	0.00000000	0.00000000	0.00000000	0.00000000	0.00000000
OTU_842 (unclassified)	0.00027560	0.00000000	0.00000000	0.00000000	0.00000000	0.00000000
OTU_843 (unclassified)	0.00013780	0.00000000	0.00000000	0.00000000	0.00000000	0.00000000
OTU_844 (unclassified)	0.00117128	0.00000000	0.00000000	0.00000000	0.00000000	0.00000000
OTU_845 (unclassified)	0.00013780	0.00000000	0.00000000	0.00000000	0.00000000	0.00000000
OTU_846 (Hyphomicrobium)	0.00020670	0.00000000	0.00000000	0.00000000	0.00000000	0.00000000
OTU_847 (unclassified)	0.00013780	0.00000000	0.00000000	0.00000000	0.00000000	0.00000000
OTU_848 (unclassified)	0.00000000	0.00000000	0.00000000	0.00043856	0.00000000	0.00031873
OTU_849 (unclassified)	0.00013780	0.00000000	0.00000000	0.00000000	0.00000000	0.00000000
OTU_850 (unclassified)	0.00034449	0.00000000	0.00000000	0.00000000	0.00000000	0.00000000
OTU_851 (Aquicella)	0.00013780	0.00000000	0.00000000	0.00000000	0.00000000	0.00000000
OTU_852 (Acinetobacter)	0.00048229	0.00081400	0.00083183	0.00080403	0.00121962	0.00071713
OTU_853 (unclassified)	0.00034449	0.00000000	0.00000000	0.00007309	0.00000000	0.00000000
OTU_854 (unclassified)	0.00027560	0.00000000	0.00000000	0.00000000	0.00000000	0.00000000

OTU_855 (unclassified)	0.00020670	0.00000000	0.00000000	0.00000000	0.00000000	0.00000000
OTU_856 (unclassified)	0.00000000	0.00097680	0.00110911	0.00021928	0.00008712	0.00366534
OTU_857 (unclassified)	0.00034449	0.00000000	0.00000000	0.00000000	0.00000000	0.00000000
OTU_858 (unclassified)	0.00020670	0.00000000	0.00000000	0.00000000	0.00000000	0.00000000
OTU_859 (Legionella)	0.00013780	0.00000000	0.00000000	0.00000000	0.00000000	0.00000000
OTU_860 (unclassified)	0.00000000	0.00097680	0.00000000	0.00007309	0.00008712	0.00000000
OTU_861 (unclassified)	0.00000000	0.00032560	0.00006932	0.00000000	0.00000000	0.00055777
OTU_862 (unclassified)	0.00000000	0.00016280	0.00000000	0.00000000	0.00000000	0.00000000
OTU_863 (unclassified)	0.00000000	0.00048840	0.00000000	0.00000000	0.00000000	0.00000000
OTU_864 (unclassified)	0.00000000	0.00097680	0.00069319	0.00131569	0.00121962	0.00135458
OTU_865 (unclassified)	0.00000000	0.00016280	0.00000000	0.00000000	0.00000000	0.00000000
OTU_866 (Flavobacterium)	0.00000000	0.00065120	0.00041592	0.00007309	0.00000000	0.00015936
OTU_867 (unclassified)	0.00000000	0.00040700	0.00000000	0.00000000	0.00000000	0.00000000
OTU_868 (Legionella)	0.00000000	0.00040700	0.00000000	0.00000000	0.00000000	0.00000000
OTU_869 (unclassified)	0.00000000	0.00032560	0.00041592	0.00007309	0.00017423	0.00015936
OTU_870 (Bryobacter)	0.00000000	0.00040700	0.00000000	0.00000000	0.00000000	0.00000000
OTU_871 (Aquiflexum)	0.00000000	0.00016280	0.00006932	0.00000000	0.00000000	0.00000000
OTU_872 (unclassified)	0.00000000	0.00000000	0.00055455	0.00065785	0.00000000	0.00000000
OTU_873 (Azospira)	0.00000000	0.00024420	0.00000000	0.00000000	0.00000000	0.00000000
OTU_874 (unclassified)	0.00000000	0.00016280	0.00027728	0.00000000	0.00000000	0.00000000
OTU_875 (unclassified)	0.00000000	0.00032560	0.00000000	0.00000000	0.00000000	0.00000000
OTU_876 (unclassified)	0.00000000	0.00000000	0.00055455	0.00000000	0.00026135	0.00000000
OTU_877 (unclassified)	0.00000000	0.00000000	0.00034660	0.00014619	0.00026135	0.00015936
OTU_878 (unclassified)	0.00000000	0.00000000	0.00027728	0.00000000	0.00017423	0.00000000
OTU_879 (unclassified)	0.00000000	0.00000000	0.00020796	0.00000000	0.00000000	0.00000000
OTU_880 (unclassified)	0.00000000	0.00048840	0.00000000	0.00000000	0.00000000	0.00000000
OTU_881 (unclassified)	0.00000000	0.00016280	0.00000000	0.00000000	0.00000000	0.00000000
OTU_882 (unclassified)	0.00000000	0.00024420	0.00000000	0.00000000	0.00000000	0.00000000
OTU_883 (unclassified)	0.00000000	0.00040700	0.00000000	0.00000000	0.00000000	0.00000000
OTU_884 (unclassified)	0.00000000	0.00024420	0.00000000	0.00000000	0.00000000	0.00000000
OTU_885 (TM7_genus_incertae_sedis)	0.00000000	0.00024420	0.00000000	0.00000000	0.00000000	0.00000000
OTU_886 (unclassified)	0.00000000	0.00179080	0.00187162	0.00190045	0.00270058	0.00350598
OTU_887 (Catenibacterium)	0.00000000	0.00016280	0.00000000	0.00000000	0.00000000	0.00000000
OTU_888 (unclassified)	0.00000000	0.00000000	0.00000000	0.00029238	0.00000000	0.00000000
OTU_889 (OD1_genus_incertae_sedis)	0.00000000	0.00032560	0.00000000	0.00000000	0.00000000	0.00000000
OTU_890 (OD1_genus_incertae_sedis)	0.00000000	0.00048840	0.00020796	0.00000000	0.00008712	0.00000000
OTU_891 (Flavobacterium)	0.00000000	0.00008140	0.00027728	0.00051166	0.00000000	0.00000000
OTU_892 (unclassified)	0.00000000	0.00056980	0.00000000	0.00007309	0.00008712	0.00000000
OTU_893 (unclassified)	0.00000000	0.00016280	0.00000000	0.00000000	0.00000000	0.00000000
OTU_894 (unclassified)	0.00000000	0.00024420	0.00000000	0.00000000	0.00000000	0.00000000
OTU_895 (Caedibacter)	0.00000000	0.00081400	0.00000000	0.00000000	0.00008712	0.00000000
OTU_896 (unclassified)	0.00000000	0.00065120	0.00020796	0.00014619	0.00008712	0.00000000
OTU_897 (unclassified)	0.00000000	0.00024420	0.00000000	0.00000000	0.00000000	0.00000000
OTU_898 (TM7_genus_incertae_sedis)	0.00000000	0.00040700	0.00000000	0.00000000	0.00000000	0.00000000
OTU_899 (unclassified)	0.00000000	0.00016280	0.00000000	0.00000000	0.00000000	0.00000000
OTU_900 (Rhizobacter)	0.00020670	0.00008140	0.00097047	0.00036547	0.00017423	0.00047809
OTU_901 (Bacillus)	0.00006890	0.00000000	0.00013864	0.00000000	0.00000000	0.00015936

OTU_902 (Achromobacter)	0.00000000	0.00105820	0.00034660	0.00036547	0.00078404	0.00023904
OTU_903 (Acinetobacter)	0.00000000	0.00000000	0.00000000	0.00007309	0.00034846	0.00023904
OTU_904 (Rubrobacter)	0.00000000	0.00024420	0.00006932	0.00000000	0.00008712	0.00015936
OTU_905 (unclassified)	0.00000000	0.00016280	0.00000000	0.00000000	0.00000000	0.00000000
OTU_906 (unclassified)	0.00000000	0.00000000	0.00020796	0.00000000	0.00000000	0.00000000
OTU_907 (unclassified)	0.00000000	0.00000000	0.00027728	0.00000000	0.00000000	0.00000000
OTU_908 (unclassified)	0.00000000	0.00008140	0.00027728	0.00007309	0.00000000	0.00000000
OTU_909 (Undibacterium)	0.00000000	0.00016280	0.00062387	0.00014619	0.00000000	0.00007968
OTU_910 (unclassified)	0.00000000	0.00000000	0.00020796	0.00000000	0.00000000	0.00000000
OTU_911 (unclassified)	0.00000000	0.00016280	0.00027728	0.00036547	0.00069692	0.00087649
OTU_912 (Polaromonas)	0.00000000	0.00000000	0.00020796	0.00000000	0.00008712	0.00015936
OTU_913 (unclassified)	0.00000000	0.00000000	0.00041592	0.00000000	0.00000000	0.00031873
OTU_914 (unclassified)	0.00000000	0.00000000	0.00000000	0.00000000	0.00095827	0.00047809
OTU_915 (unclassified)	0.00000000	0.00000000	0.00000000	0.00000000	0.00043558	0.00023904
OTU_916 (Streptococcus)	0.00000000	0.00000000	0.00055455	0.00014619	0.00000000	0.00023904
OTU_917 (unclassified)	0.00000000	0.00008140	0.00013864	0.00000000	0.00000000	0.00000000
OTU_918 (Cloacibacterium)	0.00000000	0.00008140	0.00034660	0.00007309	0.00069692	0.00031873
OTU_919 (unclassified)	0.00000000	0.00000000	0.00013864	0.00000000	0.00000000	0.00000000
OTU_920 (Lysobacter)	0.00000000	0.00000000	0.00013864	0.00014619	0.00008712	0.00007968
OTU_921 (unclassified)	0.00000000	0.00032560	0.00034660	0.00000000	0.00000000	0.00000000
OTU_922 (unclassified)	0.00000000	0.00000000	0.00041592	0.00000000	0.00000000	0.00000000
OTU_923 (unclassified)	0.00000000	0.00000000	0.00041592	0.00043856	0.00008712	0.00007968
OTU_924 (unclassified)	0.00000000	0.00008140	0.00041592	0.00000000	0.00000000	0.00015936
OTU_925 (unclassified)	0.00000000	0.00000000	0.00027728	0.00000000	0.00000000	0.00015936
OTU_926 (Luteolibacter)	0.00000000	0.00000000	0.00027728	0.00000000	0.00000000	0.00000000
OTU_927 (Mucilaginibacter)	0.00000000	0.00000000	0.00013864	0.00000000	0.00000000	0.00000000
OTU_928 (unclassified)	0.00000000	0.00000000	0.00020796	0.00000000	0.00017423	0.00031873
OTU_929 (Flectobacillus)	0.00006890	0.00000000	0.00041592	0.00007309	0.00000000	0.00000000
OTU_930 (unclassified)	0.00000000	0.00000000	0.00013864	0.00000000	0.00000000	0.00000000
OTU_931 (unclassified)	0.00000000	0.00000000	0.00020796	0.00000000	0.00000000	0.00000000
OTU_932 (unclassified)	0.00000000	0.00016280	0.00090115	0.00014619	0.00000000	0.00007968
OTU_933 (Legionella)	0.00000000	0.00000000	0.00000000	0.00000000	0.00034846	0.00000000
OTU_934 (Streptococcus)	0.00000000	0.00040700	0.00000000	0.00007309	0.00017423	0.00000000
OTU_935 (unclassified)	0.00000000	0.00065120	0.00000000	0.00014619	0.00008712	0.00007968
OTU_936 (unclassified)	0.00000000	0.00073260	0.00000000	0.00000000	0.00000000	0.00007968
OTU_937 (unclassified)	0.00027560	0.00024420	0.00000000	0.00000000	0.00000000	0.00000000
OTU_938 (unclassified)	0.00000000	0.00024420	0.00000000	0.00000000	0.00000000	0.00000000
OTU_939 (Rothia)	0.00000000	0.00016280	0.00000000	0.00000000	0.00000000	0.00000000
OTU_940 (unclassified)	0.00000000	0.00016280	0.00013864	0.00000000	0.00000000	0.00000000
OTU_941 (unclassified)	0.00000000	0.00032560	0.00000000	0.00000000	0.00000000	0.00000000
OTU_942 (unclassified)	0.00000000	0.00016280	0.00000000	0.00000000	0.00000000	0.00000000
OTU_943 (Brevundimonas)	0.00000000	0.00008140	0.00027728	0.00007309	0.00008712	0.00023904
OTU_944 (unclassified)	0.00000000	0.00032560	0.00000000	0.00000000	0.00000000	0.00000000
OTU_945 (unclassified)	0.00000000	0.00032560	0.00000000	0.00000000	0.00000000	0.00000000
OTU_946 (unclassified)	0.00000000	0.00040700	0.00000000	0.00000000	0.00000000	0.00000000
OTU_947 (unclassified)	0.00000000	0.00024420	0.00000000	0.00000000	0.00000000	0.00000000
OTU_948 (unclassified)	0.00000000	0.00032560	0.00000000	0.00000000	0.00000000	0.00007968
OTU_949 (unclassified)	0.00000000	0.00024420	0.00000000	0.00000000	0.00000000	0.00000000
OTU_950 (unclassified)	0.00000000	0.00032560	0.00006932	0.00021928	0.00026135	0.00000000
OTU_951 (unclassified)	0.00000000	0.00016280	0.00000000	0.00000000	0.00000000	0.00000000
OTU_952 (unclassified)	0.00000000	0.00000000	0.00020796	0.00000000	0.00017423	0.00000000

OTU_953 (Rothia)	0.00000000	0.00000000	0.00000000	0.00014619	0.00026135	0.00000000
OTU_954 (unclassified)	0.00000000	0.00032560	0.00055455	0.00014619	0.00000000	0.00015936
OTU_955 (unclassified)	0.00000000	0.00000000	0.00013864	0.00036547	0.00000000	0.00000000
OTU_956 (unclassified)	0.00000000	0.00024420	0.00000000	0.00000000	0.00000000	0.00000000
OTU_957 (unclassified)	0.00000000	0.00032560	0.00000000	0.00000000	0.00000000	0.00000000
OTU_958 (unclassified)	0.00000000	0.00024420	0.00020796	0.00007309	0.00000000	0.00071713
OTU_959 (Cellulosilyticum)	0.00000000	0.00032560	0.00000000	0.00000000	0.00000000	0.00000000
OTU_960 (Lactococcus)	0.00000000	0.00048840	0.00006932	0.00029238	0.00008712	0.00039841
OTU_961 (unclassified)	0.00000000	0.00024420	0.00000000	0.00000000	0.00000000	0.00000000
OTU_962 (unclassified)	0.00000000	0.00073260	0.00000000	0.00000000	0.00000000	0.00000000
OTU_963 (OD1_genus_incertae_sedis)	0.00000000	0.00032560	0.00000000	0.00000000	0.00000000	0.00000000
OTU_964 (Microbacterium)	0.00013780	0.00016280	0.00013864	0.00000000	0.00000000	0.00000000
OTU_965 (unclassified)	0.00000000	0.00073260	0.00013864	0.00007309	0.00000000	0.00000000
OTU_966 (unclassified)	0.00000000	0.00016280	0.00000000	0.00000000	0.00000000	0.00000000
OTU_967 (Leadbetterella)	0.00000000	0.00000000	0.00013864	0.00000000	0.00000000	0.00000000
OTU_968 (unclassified)	0.00000000	0.00032560	0.00000000	0.00007309	0.00017423	0.00031873
OTU_969 (unclassified)	0.00000000	0.00024420	0.00000000	0.00007309	0.00000000	0.00000000
OTU_970 (Mycobacterium)	0.00000000	0.00016280	0.00000000	0.00000000	0.00000000	0.00000000
OTU_971 (unclassified)	0.00000000	0.00032560	0.00000000	0.00000000	0.00000000	0.00000000
OTU_972	0.00000000	0.00048840	0.00000000	0.00000000	0.00017423	0.00023904
OTU_973 (Treponema)	0.00000000	0.00016280	0.00000000	0.00000000	0.00000000	0.00000000
OTU_974 (unclassified)	0.00000000	0.00040700	0.00000000	0.00000000	0.00000000	0.00000000
OTU_975 (unclassified)	0.00000000	0.00056980	0.00013864	0.00007309	0.00000000	0.00007968
OTU_976 (unclassified)	0.00000000	0.00024420	0.00000000	0.00000000	0.00000000	0.00000000
OTU_977 (unclassified)	0.00000000	0.00000000	0.00000000	0.00014619	0.00000000	0.00007968
OTU_978 (unclassified)	0.00000000	0.00065120	0.00000000	0.00000000	0.00000000	0.00000000
OTU_979 (Acinetobacter)	0.00000000	0.00065120	0.00000000	0.00043856	0.00017423	0.00007968
OTU_980 (unclassified)	0.00000000	0.00040700	0.00000000	0.00000000	0.00000000	0.00007968
OTU_981 (unclassified)	0.00000000	0.00024420	0.00000000	0.00007309	0.00000000	0.00007968
OTU_982 (Turneriella)	0.00000000	0.00016280	0.00000000	0.00014619	0.00000000	0.00000000
OTU_983 (unclassified)	0.00000000	0.00016280	0.00000000	0.00007309	0.00000000	0.00000000
OTU_984 (unclassified)	0.00000000	0.00024420	0.00000000	0.00000000	0.00000000	0.00000000
OTU_985 (unclassified)	0.00000000	0.00016280	0.00000000	0.00000000	0.00000000	0.00000000
OTU_986 (unclassified)	0.00000000	0.00024420	0.00000000	0.00000000	0.00000000	0.00000000
OTU_987 (unclassified)	0.00000000	0.00056980	0.00013864	0.00021928	0.00000000	0.00007968
OTU_988 (unclassified)	0.00000000	0.00024420	0.00000000	0.00000000	0.00000000	0.00000000
OTU_989 (unclassified)	0.00000000	0.00024420	0.00000000	0.00000000	0.00000000	0.00000000
OTU_990 (unclassified)	0.00000000	0.00032560	0.00000000	0.00000000	0.00000000	0.00000000
OTU_991 (unclassified)	0.00000000	0.00000000	0.00006932	0.00095022	0.00017423	0.00000000
OTU_992 (unclassified)	0.00000000	0.00081400	0.00020796	0.00014619	0.00008712	0.00007968
OTU_993 (Sphingopyxis)	0.00000000	0.00056980	0.00000000	0.00000000	0.00000000	0.00000000
OTU_994 (unclassified)	0.00000000	0.00048840	0.00000000	0.00000000	0.00000000	0.00000000
OTU_995 (unclassified)	0.00000000	0.00032560	0.00000000	0.00000000	0.00000000	0.00000000
OTU_996 (unclassified)	0.00000000	0.00048840	0.00000000	0.00000000	0.00000000	0.00000000
OTU_997 (unclassified)	0.00000000	0.00056980	0.00000000	0.00000000	0.00000000	0.00000000
OTU_998 (unclassified)	0.00000000	0.00024420	0.00000000	0.00000000	0.00000000	0.00000000
OTU_999 (unclassified)	0.00000000	0.00016280	0.00020796	0.00000000	0.00000000	0.00000000
OTU_1000 (Ohtaekwangia)	0.00000000	0.00048840	0.00000000	0.00000000	0.00000000	0.00000000
OTU_1001 (unclassified)	0.00000000	0.00016280	0.00000000	0.00000000	0.00000000	0.00000000
OTU_1002 (unclassified)	0.00000000	0.00040700	0.00013864	0.00000000	0.00000000	0.00000000

OTU_1003 (unclassified)	0.00000000	0.00016280	0.00000000	0.00000000	0.00000000	0.00000000
OTU_1004 (unclassified)	0.00000000	0.00024420	0.00000000	0.00000000	0.00008712	0.00000000
OTU_1005 (Legionella)	0.00000000	0.00024420	0.00000000	0.00000000	0.00017423	0.00000000
OTU_1006 (unclassified)	0.00000000	0.00000000	0.00020796	0.00000000	0.00000000	0.00000000
OTU_1007 (unclassified)	0.00000000	0.00032560	0.00000000	0.00000000	0.00008712	0.00000000
OTU_1008 (Sphingomonas)	0.00000000	0.00040700	0.00000000	0.00000000	0.00000000	0.00000000
OTU_1009 (unclassified)	0.00000000	0.00056980	0.00006932	0.00029238	0.00017423	0.00015936
OTU_1010 (unclassified)	0.00000000	0.00000000	0.00034660	0.00007309	0.00000000	0.00000000
OTU_1011 (unclassified)	0.00000000	0.00048840	0.00000000	0.00000000	0.00000000	0.00000000
OTU_1012 (Inhella)	0.00000000	0.00016280	0.00013864	0.00014619	0.00000000	0.00000000
OTU_1013 (unclassified)	0.00000000	0.00024420	0.00000000	0.00000000	0.00000000	0.00000000
OTU_1014 (unclassified)	0.00000000	0.00016280	0.00000000	0.00000000	0.00000000	0.00000000
OTU_1015 (unclassified)	0.00000000	0.00032560	0.00000000	0.00000000	0.00000000	0.00000000
OTU_1016 (unclassified)	0.00000000	0.00081400	0.00027728	0.00000000	0.00000000	0.00007968
OTU_1017 (unclassified)	0.00000000	0.00000000	0.00000000	0.00000000	0.00043558	0.00015936
OTU_1018 (unclassified)	0.00000000	0.00024420	0.00027728	0.00000000	0.00000000	0.00007968
OTU_1019 (Polaromonas)	0.00000000	0.00024420	0.00000000	0.00000000	0.00000000	0.00000000
OTU_1020 (Ferruginibacter)	0.00000000	0.00016280	0.00000000	0.00007309	0.00000000	0.00000000
OTU_1021 (unclassified)	0.00000000	0.00016280	0.00000000	0.00000000	0.00000000	0.00000000
OTU_1022 (unclassified)	0.00000000	0.00048840	0.00000000	0.00014619	0.00000000	0.00000000
OTU_1023 (unclassified)	0.00000000	0.00016280	0.00020796	0.00029238	0.00017423	0.00023904
OTU_1024 (unclassified)	0.00000000	0.00040700	0.00000000	0.00007309	0.00000000	0.00000000
OTU_1025 (unclassified)	0.00000000	0.00024420	0.00000000	0.00000000	0.00000000	0.00000000
OTU_1026 (unclassified)	0.00000000	0.00032560	0.00000000	0.00000000	0.00000000	0.00000000
OTU_1027 (Yersinia)	0.00000000	0.00032560	0.00013864	0.00000000	0.00000000	0.00007968
OTU_1028 (unclassified)	0.00000000	0.00024420	0.00000000	0.00029238	0.00008712	0.00031873
OTU_1029 (TM7_genus_incertae_sedis)	0.00000000	0.00073260	0.00000000	0.00000000	0.00000000	0.00000000
OTU_1030 (unclassified)	0.00000000	0.00008140	0.00013864	0.00000000	0.00000000	0.00007968
OTU_1031 (Methylobacterium)	0.00000000	0.00000000	0.00000000	0.00021928	0.00026135	0.00015936
OTU_1032 (unclassified)	0.00000000	0.00000000	0.00013864	0.00000000	0.00008712	0.00007968
OTU_1033 (unclassified)	0.00000000	0.00040700	0.00000000	0.00000000	0.00000000	0.00000000
OTU_1034 (unclassified)	0.00000000	0.00024420	0.00000000	0.00000000	0.00000000	0.00000000
OTU_1035 (Geobacillus)	0.00000000	0.00073260	0.00041592	0.00190045	0.00243924	0.00247012
OTU_1036 (unclassified)	0.00000000	0.00000000	0.00000000	0.00000000	0.00043558	0.00031873
OTU_1037 (unclassified)	0.00000000	0.00000000	0.00020796	0.00000000	0.00000000	0.00000000
OTU_1038 (unclassified)	0.00000000	0.00000000	0.00013864	0.00000000	0.00000000	0.00000000
OTU_1039 (unclassified)	0.00000000	0.00032560	0.00000000	0.00000000	0.00000000	0.00000000
OTU_1040 (unclassified)	0.00000000	0.00056980	0.00000000	0.00000000	0.00000000	0.00000000
OTU_1041 (unclassified)	0.00000000	0.00073260	0.00000000	0.00000000	0.00000000	0.00000000
OTU_1042 (unclassified)	0.00000000	0.00016280	0.00000000	0.00000000	0.00000000	0.00000000
OTU_1043 (unclassified)	0.00000000	0.00016280	0.00000000	0.00000000	0.00000000	0.00000000
OTU_1044 (Brevundimonas)	0.00020670	0.00000000	0.00055455	0.00021928	0.00017423	0.00039841
OTU_1045 (unclassified)	0.00000000	0.00000000	0.00000000	0.00007309	0.00000000	0.00015936
OTU_1046 (unclassified)	0.00000000	0.00016280	0.00013864	0.00000000	0.00000000	0.00000000
OTU_1047 (unclassified)	0.00000000	0.00040700	0.00000000	0.00000000	0.00000000	0.00000000
OTU_1048 (unclassified)	0.00000000	0.00048840	0.00000000	0.00000000	0.00000000	0.00000000
OTU_1049 (Leadbetterella)	0.00000000	0.00016280	0.00041592	0.00000000	0.00008712	0.00015936
OTU_1050 (unclassified)	0.00000000	0.00032560	0.00000000	0.00000000	0.00000000	0.00000000
OTU_1051 (Methylotenera)	0.00000000	0.00056980	0.00000000	0.00000000	0.00000000	0.00000000
OTU_1052 (unclassified)	0.00000000	0.00016280	0.00000000	0.00000000	0.00000000	0.00000000

OTU_1053 (TM7_genus_incertae_sedis)	0.00000000	0.00040700	0.00013864	0.00000000	0.00000000	0.00000000
OTU_1054 (unclassified)	0.00000000	0.00000000	0.00041592	0.00000000	0.00008712	0.00000000
OTU_1055 (Methylobacterium)	0.00000000	0.00000000	0.00027728	0.00007309	0.00008712	0.00007968
OTU_1056 (unclassified)	0.00000000	0.00000000	0.00020796	0.00000000	0.00000000	0.00000000
OTU_1057 (Flavobacterium)	0.00000000	0.00040700	0.00055455	0.00021928	0.00000000	0.00007968
OTU_1058 (unclassified)	0.00000000	0.00016280	0.00041592	0.00000000	0.00008712	0.00031873
OTU_1059 (Clostridium_XI)	0.00000000	0.00000000	0.00000000	0.00021928	0.00043558	0.00000000
OTU_1060 (Microlunatus)	0.00000000	0.00000000	0.00020796	0.00000000	0.00017423	0.00007968
OTU_1061 (unclassified)	0.00000000	0.00000000	0.00034660	0.00000000	0.00008712	0.00007968
OTU_1062 (Geobacillus)	0.00000000	0.00000000	0.00006932	0.00014619	0.00034846	0.00007968
OTU_1063 (unclassified)	0.00000000	0.00000000	0.00006932	0.00000000	0.00226501	0.00000000
OTU_1064 (unclassified)	0.00000000	0.00000000	0.00013864	0.00000000	0.00000000	0.00000000
OTU_1065 (unclassified)	0.00000000	0.00000000	0.00041592	0.00051166	0.00000000	0.00007968
OTU_1066 (unclassified)	0.00000000	0.00000000	0.00020796	0.00000000	0.00000000	0.00015936
OTU_1067 (unclassified)	0.00000000	0.00000000	0.00020796	0.00000000	0.00000000	0.00015936
OTU_1068 (unclassified)	0.00000000	0.00000000	0.00013864	0.00000000	0.00000000	0.00000000
OTU_1069 (unclassified)	0.00000000	0.00000000	0.00034660	0.00000000	0.00000000	0.00000000
OTU_1070 (Nubsella)	0.00000000	0.00000000	0.00055455	0.00043856	0.00008712	0.00015936
OTU_1071 (unclassified)	0.00000000	0.00000000	0.00041592	0.00000000	0.00000000	0.00007968
OTU_1072 (unclassified)	0.00000000	0.00000000	0.00020796	0.00007309	0.00000000	0.00015936
OTU_1073 (Ferruginibacter)	0.00000000	0.00000000	0.00013864	0.00000000	0.00000000	0.00000000
OTU_1074 (SR1_genus_incertae_sedis)	0.00000000	0.00000000	0.00027728	0.00000000	0.00000000	0.00000000
OTU_1075 (unclassified)	0.00000000	0.00008140	0.00041592	0.00080403	0.00026135	0.00007968
OTU_1076 (unclassified)	0.00000000	0.00000000	0.00027728	0.00000000	0.00000000	0.00000000
OTU_1077	0.00000000	0.00065120	0.00000000	0.00000000	0.00000000	0.00007968
OTU_1078 (unclassified)	0.00000000	0.00008140	0.00062387	0.00087713	0.00000000	0.00000000
OTU_1079 (unclassified)	0.00000000	0.00000000	0.00034660	0.00000000	0.00000000	0.00015936
OTU_1080 (unclassified)	0.00000000	0.00000000	0.00041592	0.00029238	0.00017423	0.00007968
OTU_1081 (unclassified)	0.00000000	0.00000000	0.00013864	0.00000000	0.00000000	0.00000000
OTU_1082 (unclassified)	0.00000000	0.00040700	0.00055455	0.00014619	0.00000000	0.00023904
OTU_1083 (unclassified)	0.00000000	0.00000000	0.00055455	0.00000000	0.00000000	0.00023904
OTU_1084 (unclassified)	0.00000000	0.00016280	0.00020796	0.00007309	0.00026135	0.00007968
OTU_1085 (unclassified)	0.00000000	0.00000000	0.00034660	0.00000000	0.00000000	0.00000000
OTU_1086 (Zoogloea)	0.00000000	0.00040700	0.00013864	0.00000000	0.00000000	0.00000000
OTU_1087 (Microbacterium)	0.00000000	0.00000000	0.00041592	0.00000000	0.00000000	0.00000000
OTU_1088 (Leptotrichia)	0.00000000	0.00008140	0.00034660	0.00000000	0.00026135	0.00015936
OTU_1089 (unclassified)	0.00000000	0.00000000	0.00000000	0.00000000	0.00017423	0.00000000
OTU_1090 (unclassified)	0.00000000	0.00000000	0.00020796	0.00014619	0.00008712	0.00015936
OTU_1091 (Streptococcus)	0.00000000	0.00040700	0.00000000	0.00021928	0.00017423	0.00015936
OTU_1092 (unclassified)	0.00013780	0.00000000	0.00020796	0.00095022	0.00008712	0.00007968
OTU_1093 (Mycobacterium)	0.00000000	0.00008140	0.00027728	0.00000000	0.00000000	0.00015936
OTU_1094 (Veillonella)	0.00027560	0.00000000	0.00048523	0.00007309	0.00000000	0.00000000
OTU_1095 (unclassified)	0.00000000	0.00000000	0.00013864	0.00014619	0.00000000	0.00015936
OTU_1096 (unclassified)	0.00000000	0.00000000	0.00020796	0.00000000	0.00000000	0.00047809
OTU_1097 (unclassified)	0.00000000	0.00000000	0.00013864	0.00000000	0.00000000	0.00000000
OTU_1098 (Leadbetterella)	0.00000000	0.00000000	0.00027728	0.00007309	0.00000000	0.00015936
OTU_1099 (unclassified)	0.00000000	0.00000000	0.00020796	0.00000000	0.00000000	0.00000000
OTU_1100 (OD1_genus_incertae_sedis)	0.00000000	0.00008140	0.00020796	0.00000000	0.00000000	0.00000000

OTU_1101 (unclassified)	0.00000000	0.00000000	0.00034660	0.00036547	0.00000000	0.00031873
OTU_1102 (unclassified)	0.00000000	0.00000000	0.00013864	0.00021928	0.00000000	0.00000000
OTU_1103 (unclassified)	0.00000000	0.00000000	0.00048523	0.00000000	0.00017423	0.00031873
OTU_1104 (unclassified)	0.00000000	0.00000000	0.00034660	0.00014619	0.00060981	0.00015936
OTU_1105 (unclassified)	0.00000000	0.00000000	0.00020796	0.00000000	0.00000000	0.00007968
