# Supplementary material for: Effects of Host Phylogeny and Habitats on Gut Microbiomes of Oriental River Prawn (Macrobrachium nipponense)
Source: PLoS One. 2015 Jul 13;10(7):e0132860. doi: 10.1371/journal.pone.0132860 (PMC4500556; doi:10.1371/journal.pone.0132860)
Supplement: S3 Table — (DOCX) [file pone.0132860.s004.docx]

**S3 Table. Weight distribution of collected shrimp.**

| Location | Species | Lineage | Weight(g) | Sex |
| --- | --- | --- | --- | --- |
| Chishan River | *Macrobrachium asperulum* | - | 2.7 | ♂ |
| Chishan River | *Macrobrachium asperulum* | - | 2.5 | ♂ |
| Chishan River | *Macrobrachium asperulum* | - | 2.5 | ♂ |
| Chishan River | *Macrobrachium asperulum* | - | 2.4 | ♂ |
| Chishan River | *Macrobrachium asperulum* | - | 4.4 | ♂ |
| Chishan River | *Macrobrachium nipponense* | Chishan | 3.3 | ♂ |
| Chishan River | *Macrobrachium nipponense* | Chishan | 2.7 | ♂ |
| Chishan River | *Macrobrachium nipponense* | Chishan | 2.1 | ♂ |
| Chishan River | *Macrobrachium nipponense* | Chishan | 1.9 | ♂ |
| Chishan River | *Macrobrachium nipponense* | Chishan | 2.5 | ♂ |
| Tahan River | *Macrobrachium nipponense* | Chishan | 1.7 | ♂ |
| Tahan River | *Macrobrachium nipponense* | Chishan | 2.1 | ♂ |
| Tahan River | *Macrobrachium nipponense* | Chishan | 1.7 | ♂ |
| Tahan River | *Macrobrachium nipponense* | Chishan | 2.5 | ♂ |
| Tahan River | *Macrobrachium nipponense* | Chishan | 2 | ♂ |
| Mingte Reservoir | *Macrobrachium nipponense* | Chishan | 2.8 | ♂ |
| Mingte Reservoir | *Macrobrachium nipponense* | Chishan | 2.7 | ♂ |
| Mingte Reservoir | *Macrobrachium nipponense* | Chishan | 2.4 | ♂ |
| Mingte Reservoir | *Macrobrachium nipponense* | Chishan | 2.3 | ♂ |
| Mingte Reservoir | *Macrobrachium nipponense* | Chishan | 2.5 | ♂ |
| Mingte Reservoir | *Macrobrachium nipponense* | Shihmen | 3.2 | ♂ |
| Mingte Reservoir | *Macrobrachium nipponense* | Shihmen | 2.5 | ♂ |
| Mingte Reservoir | *Macrobrachium nipponense* | Shihmen | 2.9 | ♂ |
| Mingte Reservoir | *Macrobrachium nipponense* | Shihmen | 2.3 | ♂ |
| Mingte Reservoir | *Macrobrachium nipponense* | Shihmen | 1.9 | ♂ |
| Shihmen Reservoir | *Macrobrachium nipponense* | Shihmen | 2.1 | ♂ |
| Shihmen Reservoir | *Macrobrachium nipponense* | Shihmen | 2.4 | ♂ |
| Shihmen Reservoir | *Macrobrachium nipponense* | Shihmen | 2.8 | ♂ |
| Shihmen Reservoir | *Macrobrachium nipponense* | Shihmen | 2.7 | ♂ |
| Shihmen Reservoir | *Macrobrachium nipponense* | Shihmen | 2.5 | ♂ |
