# Supplementary material for: Effects of Host Phylogeny and Habitats on Gut Microbiomes of Oriental River Prawn (Macrobrachium nipponense)
Source: PLoS One. 2015 Jul 13;10(7):e0132860. doi: 10.1371/journal.pone.0132860 (PMC4500556; doi:10.1371/journal.pone.0132860)
Supplement: S4 Table — (DOCX) [file pone.0132860.s005.docx]

**S4 Table. Primer list of 16S rRNA gene amplicon preparation.**

| **Primer Name** | **Sequence (5'->3') (Lib-L A Adaptor+key+MID1+specific primer)** | **Length** |
| --- | --- | --- |
| A-27F_MD1 | **ccatctcatccctgcgtgtctccgac** **tcag ACGAGTGCGT AGAGTTTGATCM TGGCTCAG** | 60 |
| A-27F_MD2 | **ccatctcatccctgcgtgtctccgac** **tcag ACGCTCGACA AGAGTTTGATCM TGGCTCAG** | 60 |
| A-27F_MD3 | **ccatctcatccctgcgtgtctccgac** **tcag AGACGCACTC AGAGTTTGATCM TGGCTCAG** | 60 |
| A-27F_MD4 | **ccatctcatccctgcgtgtctccgac** **tcag AGCACTGTAG AGAGTTTGATCM TGGCTCAG** | 60 |
| B-355R | cctatcccctgtgtgccttggcagtc**tcag**GCTGCCTCCCGTAGGAGT | 48 |
